# Video, text, and memory: An emotional verbal overshadowing effect

**DOI:** 10.3758/s13421-025-01840-8

**Published:** 2026-07-02

**Authors:** Heath A. Demaree, Rock Lim, Amanda R. Merner, Caitlyn N. Dahnke, Gabriel A. Radvansky

**Affiliations:** 1 Case Western Reserve University, Cleveland, OH USA; 2 University Medical Center Göttingen, Göttingen, Germany; 3 Harvard University, Cambridge, MA USA; 4 Department of Psychology, University of Notre Dame, Notre Dame, IN 46556 USA

**Keywords:** Video memory, Event cognition, emotion

## Abstract

The present research was designed to determine whether an ambiguous, visually presented event is better recalled if an emotional (relative to neutral) verbal interpretation of the event is read before or after seeing the video. There are two competing hypotheses. First, researchers have found that emotional events are better recalled relative to neutral events. As such, one possibility is that the presentation of an emotional verbal interpretation of the event – read before or after the video itself – may enhance subsequent memory of the event. Alternatively, research on the “verbal overshadowing effect” shows that the subsequent verbal description of an event can impair memory for the event itself. This suggests that information presented asynchronously to the video may adversely affect memory for the video. We showed participants (*N* = 649) 2-min videos that could be interpreted in either a mildly or a very negative emotional way. Before or after viewing a video, people were given a script that allowed for a neutral or negative verbal interpretation of the video itself, with the negative interpretation causing them to have a more robust emotional response to the video. Memories of the video were then assessed either immediately or following a 1- or 7-day delay. Memory of both the video (using detail, inference, and wrong probes) and the text (using verbatim, paraphrase, inference, and wrong probes) were examined. Results revealed an “emotional verbal overshadowing effect,” such that emotional information presented asynchronously to the video produced the greatest decrement in subsequent memory.

## Introduction

Memory for events is prone to a number of different influences, including emotion, reinterpretation, and other post-encoding information. The aim of the current study was to explore whether memory for an experienced event can be improved, altered, or impaired by additional emotional content apart from the event itself. We also manipulated whether that additional information came before or after the critical event. Although event memories are durable, some aspects of those memories may be more or less prone to external influences, depending on the role they play in memory and cognition.

A well-established finding is that people remember emotional events better than neutral experiences (e.g., Hamann, 2001; Kensinger, 2009; LaBar & Phelps, 1998; Nadel & Jacobs, 1998; Talmi, 2013). This is driven more by the intensity of the emotion than by whether it is positive or negative (Talarico et al., 2004). Emotional experiences trigger increased activation of the amygdala and hippocampus, neurological structures important to memory formation (Davis, 1997; Mishkin & Appenzeller, 1987). That said, potential benefits for emotion on memory may not be observed immediately, but after a delay – such as an hour, a day, or a week (e.g., Anderson et al., 2006; Kleinsmith & Kaplan, 1963). This boost applies to both the emotional content itself as well as associated, separate information that may be learned around the same time (Kleinsmith & Kaplan, 1963; 1964).

Perhaps the clearest example of this is with flashbulb memories (Brown & Kulik, 1977; Hirst & Phelps, 2016). These are memories for surprising, emotional events, as well as, importantly, the circumstances in which that information is learned. Many studies of flashbulb memories assess memories for information such as where people were when they learned of the event, who they were with, how they learned of the event, what were they wearing, and so on. What is striking in these cases is the high level of detail reported for not only the event itself, but for other information that was associated with the event, in terms of people’s experiences unrelated to the emotional content, per se. This is a case in which emotional content spills over to improve memory for otherwise neutral content.

We also, however, understand that flashbulb memories are prone to error and may change over time (McCloskey et al., 1988; Niedźwieńska, 2003; Schmolck et al., 2000; Talarico & Rubin, 2003). That said, those events, as well as the neutral circumstances in which news events were learned, are more accurately remembered than other events from around the same time, and tend to be more stable over time (Cordonnier & Luminet, 2021; Kvavilashvili et al., 2009). Thus, overall, the emotional content does improve memory, even if it is not perfect.

While there is some suggestion that emotional content may boost some associated memories, there is also some evidence that it may alter our understanding of what happened, particularly when it follows the experience of the original event. Some lines of research show that when people are presented with additional information, they may then alter their understanding of the original event to have the memory conform to the new information (Hamm & Hasher, 1992; Zacks, Hasher, Doren, Hamm, & Attig, 1987). A classic example of this involves the presentation of misleading post-event information (e.g., Loftus, 1979; Loftus & Hoffman, 1989; Zaragoza & Koshmider, 1989). In these studies, people witness an event, such as a robbery or car accident. Then, afterward, they encounter additional, incorrect information. What happens is that memory for the original event may now be distorted to conform to the misleading information encountered later. As such, it is possible that learning additional information about a previously experienced event may alter the prior memory. However, in these cases, the memories are altered by what followed the original event. In light of this, the current study was designed, in part, to assess how information presented before or after an event may influence encoding and recall of the event itself.

People tend to describe their own perspectives and interpretations when discussing events. Therefore, we often hear different descriptions of the same events from different people. This is useful to us because we may not have been attending to everything, and there may have been other things that happened that we were not aware of or have been otherwise unable to learn or notice. Thus, these additional descriptions can alter and expand our memories of the events that we witnessed.

A research finding on memory that is important in this regard is verbal overshadowing (e.g., Chin & Schooler, 2008; Meissner & Brigham, 2001; Schooler & Engstler-Schooler, 1990). Here, memory is worse if people provide verbal descriptions of an event after it has been witnessed. Essentially, memory for the verbal description serves to impair the memory for some aspect of the event itself. Often, this description refers to memory for a person’s face. In essence, when there is a verbal description related to witnessed events, then the memory for the verbal description can compete with the event memory for what was actually seen.

Finally, an important, often-overlooked point about episodic event memories is that there are multiple types of information and levels of memory that can influence recall. One common distinction that is made is between detail and gist information. Detail information generally refers to precise information that was present at the time of encoding. In comparison, gist information refers to a more general understanding of the event that was encountered, including inferences that may have been drawn to facilitate understanding. Different levels of information may have different qualities. For example, detail information tends to be forgotten more rapidly than gist information about an event (Fisher & Radvansky 2018).

In terms of the level of representation and emotion, the evidence is mixed. In some cases, it appears that negative emotional content can influence detail and gist memories in different ways. Specifically, detail memory is hurt by emotional content (Subramanian et al., 2014), whereas gist memories are helped (Adolphs et al., 2005; Burke et al., 1992). This may be because, for negative emotional materials, people rely more on familiarity information (Bookbinder & Brainerd, 2016).^1^ Part of this may also reflect tunnel memory in which there is better memory for central details as compared to peripheral details (Christianson & Loftus, 1991). Thus, in addition to better overall memory for gist over detailed information, we may see this difference magnified by negative, rather than neutral, content.

The current study aimed to assess whether emotional, versus neutral, text read prior to or after watching a video would influence later memory for what was seen, and whether this influence changed over time. To this end, we manipulated (a) the delay between viewing the videos and reading the texts, and the administering of the memory assessments, (b) whether the text came before or after watching the video, and (c) the nature of the text itself, whether it was more neutral or emotionally intense in content. Importantly, the additional (neutral or negative) script included information about events that happened subsequent to the film itself. For example, one script said that two mildly combative people in the film ultimately formed a mutual understanding (yielding a neutral interpretation of the film) whereas the negative script said that one person in the film subsequently murdered the other (yielding a negative interpretation). As such, the *content* of the film was not manipulated in any way but, rather, the emotional interpretation of the event was (i.e., it was or was not part of an escalating situation). We believe that this has face validity: Can eye-witnesses reliably testify about an interaction seen between two people at a park when they later learn that ultimately led to a horrific event?

With regard to delay, we expected memory to grow worse over time (Ebbinghaus, 1885), although we did not have any strong predictions regarding differences in the patterns of forgetting over time. That said, some work has shown that differences between conditions can change over time. For example, some effects that are absent initially may emerge after a day or a week (e.g., Anderson et al., 2006; Kleinsmith & Kaplan, 1963; Roediger & Karpicke, 2006), whereas others may be present initially but become absent after a similar amount of time (Staugaard & Berntsen, 2019). Consistent with this as well as other work showing that such changes can occur over the period of a week (e.g., Fisher & Radvansky, 2018), we tested memory later in the same session, 1 day later, and after 7 days.

With regard to text position, it seems almost intuitive that the content of texts read prior to viewing a video will color one’s interpretation of it by setting up expectations. The interesting question is what happens for text that follows it. One possibility is that memory for the video, having been established prior to reading the texts, will be largely unaffected by text valence. Another is that, consistent with work on altered interpretations (Hamm & Hasher, 1992; Zacks et al., 1987), memory for the video will be improved when it is followed by supplemental text content. Finally, a third possibility is that, consistent with research on verbal overshadowing (Chin & Schooler, 2008; Meissner & Brigham, 2001; Schooler & Engstler-Schooler, 1990), memory for the video content will be worse when followed by a text.

With regard to text emotionality, we expected our emotional text versions to have a greater influence on memory than neutral text versions. If there is a spill-over influence, as is seen with work on flashbulb memories, then we would expect memory for the video context to be improved by reading more emotional accompanying texts. However, if the more emotional information draws attention to itself, then we would expect more emotional texts to be accompanied by worse memory for the content of the videos.

Of further interest is how the emotional content of the text affects memory for the text itself, as well as how this interacts with its position, in terms of memory for the video content. Overall, there is an expectation that memory for the text will be better when it is more negative than neutral. Moreover, it is expected that, whatever impact the presence of text has on memory for the video, either before or after viewing, these influences will be magnified when the text is more emotionally charged. Although we were primarily interested in learning how subsequently presented verbal information may impact prior recall of a watched event, we presented non-emotional and emotional scripts both prior to and after the film to assess how they differentially impact film recall while keeping the overall amount of script information constant.

Over and above these measures of memory per se, as an exploratory component, we also assessed participants’ confidence in their memories. This was done because memory confidence ratings can provide insight into memory processes when they vary (e.g., Odinot & Wolters, 2006; Robinson & Johnson, 1996; Wixted & Wells, 2017). While there are meaningful deviations, by and large there is a fairly good positive relationship between confidence and memory accuracy. There was a general expectation that memory would decline over time, consistent with prior work (Ebbinghaus, 1885/1964; Radvansky, 2022, 2024; Rubin & Wenzel, 1996; Wixted & Ebbesen, 1991). However, it was unclear if the nature of the additional verbal information would differentially influence confidence ratings in our various conditions.

In addition to the more memory focused aspects of the study, as an exploratory step, we also assessed how our manipulations influenced attitudes related to the content of the videos and texts. In particular, participant attitudes were assessed that concerned the viewers’ respect for others, self-control, appreciation that people need to think before they act, as well as the awareness that our actions have consequences. This was done with the idea that the contents of memory from the verbal descriptions could modify not only what people remember, but also influence the attitudes participants have, and how these influences change over time. The change in attitudes over time has been underexplored in the literature, which typically focuses on attitudes after a single timepoint (e.g., Maio et al., 2019.).

## Pilot study

The pilot study was designed to identify videos and texts that were suitable for use in the main study. Specifically, our aim was to select videos that were engaging and that had some negative emotional component which could then be amplified by the text when appropriate. Our expectation was that the videos would induce a negative emotion in the participants even when a neutral interpretation of the video was provided, but the negative emotional response to the films would be potentiated when the participant was provided with the negative emotional text.

### Method

#### Participants

This study included 1,909 participants, aged 18 years or older, recruited via mTurk, who agreed to our Informed Consent Form. They self-declared where they were living, and they were excluded from participation if they lived outside of the USA (*N* = 297) so that there would be a common cultural perspective when viewing the videos. As part of a prescreening, the rest answered 17 true/false questions. For each question, a person was asked to “please indicate whether you have strong emotional reactions (e.g., you become sweaty or shaky, have noticeable increases in heart rate, etc.) to” 17 different items (e.g., going to the dentist, looking down from high altitudes). A critical question was whether the participant endorsed “true” to “hearing about an act of violence.” As requested by our Institutional Review Board, participants were not allowed to participate if they endorsed this question (*N* = 1,114). Also embedded into this questionnaire were three attention checks. Data were excluded if any of the attention checks were failed (*N* = 88). Lastly, data were excluded if they watched the videos for less than 120 s (*N* = 24), indicating that they had fast-forwarded one or more of the videos. Lastly, data from 107 participants were excluded for failure to complete the experiment. As such, the final number of participants in this study was 297. There were 146 viewers for the Lyft video and 151 viewers for the City Council meeting video. This study was approved by the Case Western Reserve University Institutional Review Board.

#### Materials

We identified two videos from youtube.com that were potentially worthy for use in the main study. Both videos were slightly trimmed to be 2 min in duration. One of these was about an experience in a rideshare (Lyft) car (https://www.youtube.com/watch?v=oACyjoLtU6E), whereas the other video was about a City Council meeting (https://www.youtube.com/watch?v=58oR7gjqwQE).

We also created neutral and emotional (negative) text to accompany the videos. These texts were designed to alter the interpretation of the videos in a relatively neutral or negative way and were presented either before or after the videos. This text was presented as a description of events that happened after the event shown in the video. There were two versions of each text for a given video. One was a neutral version that described events that were largely emotionally neutral, if not slightly emotionally positive, to try to attenuate the slightly negative nature of the video. The other was a more emotional version that provided an interpretation of the video in a very negative manner. All videos and texts are available on-line at: https://osf.io/qbw23/

#### Procedure

After informed consent and questionnaires, participants next completed the Self-Assessment Manikin (SAM).^2^ The SAM assessed each participant’s valence (from 1 to 9, very negative to very positive) at the moment. Next, each participant watched a video (2: Driver vs. City Council) with Text Valence (2: Neutral vs. Negative) presented at different times (or “Text Positions”) (2: before the video vs. after). Last, they once again completed the SAM to assess their emotional valence after the video/text. At the end of the study, participants were informed that the text information provided was fictitious, and that the researchers had no knowledge of what transpired after the videos.

## Results

To ensure that we selected neutral to mildly negative videos, we first examined the emotional responses participants reported to each video (2: Lyft vs. Meeting) in response to the Neutral Text read at one of two different times (Before vs. After) relative to the video. Emotional response data was collected using the SAM Valence scale (Likert scale from 1 (very negative) to 5 (neutral) to 9 (very positive)). Emotional Valence was always modestly less (more negative) than 5 (“Neutral”): Meeting video read Before (*N* = 33, *M* = 3.33, 95% *CI* = 2.76–3.91); Meeting video read After (*N* = 39, *M* = 4.09, 95% *CI* = 3.47–4.72); Lyft video read Before (*N* = 36, *M* = 2.35, 95% *CI* = 1.76–2.96); Lyft video read After (*N* = 36, *M* = 3.22, 95% *CI* = 2.63–3.82).

**Fig. 1:**
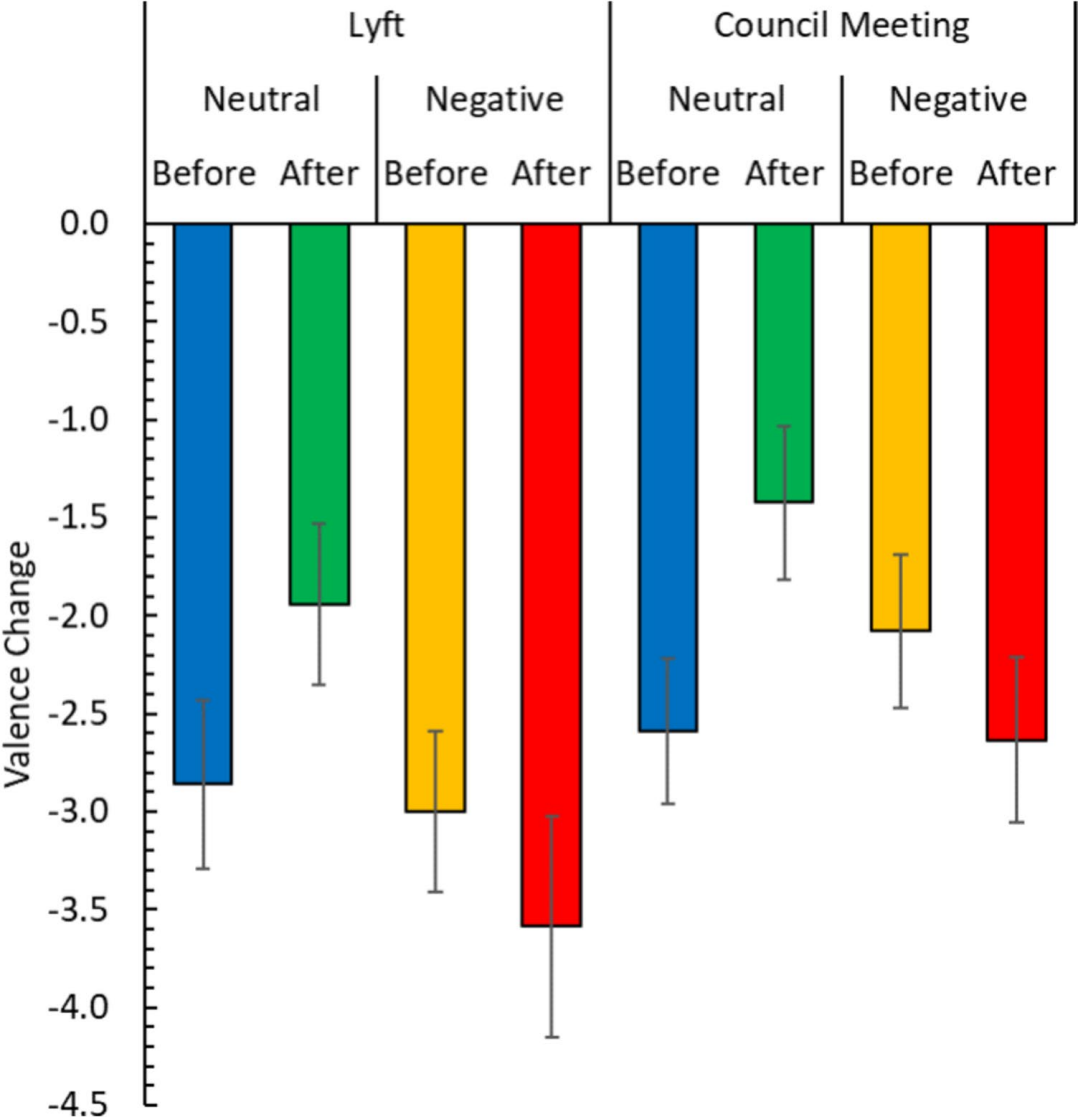
SAM Valence 2 – Same Valence 1 by video, text type, and relative time

Next, to measure affective *change* to the video (2: Lyft vs. Meeting), Text Valence (2: Neutral vs. Negative) and Text Time (2: Before vs. After), our dependent variable was change in valence as measured by the Time 2 SAM Valence ratings minus the Time 1 SAM Valence ratings.^3^ See Fig. 1 for a breakdown of valence change scores.

Valence reactivity data were submitted to a 2 (Video: Driver vs. City Council) x (Position: Before or After) X 2 (Text Valence: Neutral or Negative) ANOVA, with all variables being between-subjects. Overall, there was a main effect of Video, *F*(1,296) = 4.89,* p* =.03, *η*_*p*_^*2*^ =.017, with the Driver video inducing greater negative change in valence (*M* = −2.84; *SE* =.23) than the City Council video (*M* = −2.34; *SE* =.27), although the overall magnitude of this difference between these videos was modest.

There was also a main effect of Text Valence, *F*(1,296) =4.23,* p* =.04, *η*_*p*_^*2*^ =.014, with the Negative text inducing greater negative change in emotion in viewers than the Neutral text. Not surprisingly, negative content made people feel a bit worse. The effect of Text Position was not significant, *F* < 1.

In addition to the main effects, there was a significant Text Valence x Position interaction, *F*(1,296) = 7.15,* p* =.008, *η*_*p*_^*2*^ =.024. Regardless of the video viewed, simple effects showed that, whereas the Neutral text had a greater impact on emotion when read before relative to after the video, *F*(1,142) = 6.53,* p* <.02, *η*_*p*_^*2*^ =.044, the Negative text had equivalent effects when read either before or after the video, *F*(1,151) = 1.32,* p* <.26, *η*_*p*_^*2*^ =.009. No other interactions were significant, all *p*s >.30.

In sum, the pilot study established that the videos and text did influence affective experience. As such, we were confident that they would be appropriate for the memory assessment of the main study.

## Main study

In the main study, people saw two videos and were given one of two texts describing the events seen. These texts either provided additional information that was (a) more emotionally neutral or (b) more emotionally negative. The text was presented either before or after viewing the video. For this study, the emotional text was always presented for the second video because, if it was presented to the first video, it could color the viewing and interpretation of the second one. Because of verbal overshadowing, the hypothesis was that detail memory for the video would be worse when the text came after the video than before, and that this disruption would be greater when the text was negative compared to neutral. This is because a negative text would draw attention away from the video memory. Although there are no strong a priori theoretical predictions, we suspected that this may have a strong influence on detail memory of the video compared to more general gist memory of it.

Moreover, for text memory, it was expected that its occurrence before or after the video would have no impact. However, memory for the text would be better for the negative than the neutral version. There are no clear a priori predictions for how this would affect the different levels of representation of the text.

Given that memory changes over time (Ebbinghaus, 1885), and that differences between levels of memory can sometimes emerge over a 1-week period (e.g., Fisher & Radvansky, 2018), we assessed memory for the videos and the texts immediately, after 1 day, or 1 week later.

Finally, as an exploratory measure, we assessed attitudes as they related to the videos, and how they were affected by the different texts. Moreover, we also assessed confidence. The attitude questions were related to “gist memory,” but designed to investigate general lessons learned or inferred from the Video/Text. In short, these questions were designed to determine whether participants learned any “life lesson” from the video, even if they did not remember the specifics of what they had seen or read. Confidence was assessed because some literature suggests that flashbulb memories are associated with a person having great confidence in what they remember, although their recollection of the event may, in fact, be false.

### Method

#### Participants

For the experiment, 649 participants were recruited from the participant pool in the Department of Psychology at the University of Notre Dame. These data were collected on-line. There were 241 people tested in the immediate testing group, 192 in the 1-day delay group, and 216 in the 7-day delay group. This was based on a target of 192 participants in each retention group as determined using G*Power to observe a medium effect with our experimental design. These participants were compensated with partial course credit. The data from 40 participants (6.9% of the data) were dropped for incorrectly identifying greater than 40% of the incorrect probes across both the videos and the texts, indicating poor attention and processing of the materials. This included 13 participants from the immediate testing group, 11 participants from the 1-day testing group, and 16 participants from the 7-day testing group.

#### Materials

The experiment used the videos and texts that were assessed in the pilot study. In addition, a number of items were created to assess memory and attitudes in response to the videos and texts. We assessed memory for both the video itself, as well as the text that accompanied the video. Three types of memory probes were used to assess memory of specific types of information. The first, *detail probes*, referred to the visual elements of the video. Each probe required a yes or no response. An example of one memory probe was “The car driver was wearing a gray t-shirt.” The aim of these questions was to tap into detail memory for the video itself. A second probe type, *inference probes*, was aimed at viewers’ understanding beyond what was in the video itself. That is, they tapped into their event models of the video by assessing inferences that were likely to be drawn when watching. An example is, “It was a warm night out.” In addition to the detail and inference probes, there were also *wrong probes* that conveyed information that was inconsistent with what happened in the video. An example of one of these was, “The car driver was wearing a blue sweater.” There were four probes for each of the three probe types for each video.

In terms of the text, the Schmalhofer-Glavanov (1986) method was used to assess three levels of text memory, namely the surface form, the textbase, and the event model. The surface form reflects memory for the precise wording of a text. This level of memory is generally very poor, and forgetting tends to be quite rapid (Sachs, 1967). The textbase is memory for the propositional idea units present in the text, apart from the actual wording. This information is remembered better than the surface form, but is still forgotten at a fairly steady rate. Finally, the event model is a memory for the events described by the text. As such, it is not a memory for the text itself, but a memory for what the text is about. Thus, it is a referential level of memory (Glenberg, Meyer, & Lindem, 1987).

For the text analysis, there were four probe types for a given target sentence: *verbatim*, *paraphrase*, *inference*, and *wrong*. Verbatim probes were sentences that were actually present in the text. An example of one of these memory probes was, “The original driver gave warnings through the app about these passengers’ behaviors.” Paraphrases were rewordings of sentences from the text. An example of one of these memory probes was, “Through the app, the first driver gave warnings about the passengers’ behaviors.” Inferences were information that was not in the text, but were consistent with the situations described by the texts, and were likely drawn by readers during comprehension. An example of one of these memory probes was, “The original driver wanted to block these passengers from getting a ride with someone else.” Finally, wrong probes were thematically consistent with the text, but were inconsistent with the situation described by the text. An example of one of these memory probes was, “The original driver did not give a warning about these passengers’ behaviors.”

For the Schmalhofer-Glavanov (1986) analysis, signal detection measures are used to calculate indices of the surface form, textbase, and event model levels. For the surface form level, “yes” responses to verbatim probes are treated as hits, and “yes” responses to paraphrases are treated as false alarms. Here, both probe types convey information that was actually in the text, but only the verbatim probes capture the exact wording. For the textbase level, “yes” responses to paraphrases are treated as hits, and “yes” responses to inferences are treated as false alarms. Here, both probe types convey information that is consistent with the text, but only the paraphrase probes capture information that was actually in the text. For the event model level, “yes” responses to inferences are treated as hits, and “yes” responses to wrongs are treated as false alarms. Here, both probe types are thematically consistent with the text but only the inference probes capture what was the text was about. This approach to assessing memory has been used repeatedly in the literature (Bohay, Blakely, Tamplin, & Radvansky, 2011; Chronister, Tamplin, & Radvansky, 2022; Fisher & Radvansky, 2018; Fletcher & Chrysler, 1990; Kintsch, Welsch, Schmalhofer, & Zimny, 1990; Narvaez, Radvansky, Lynchard, & Copeland, 2011; O’Rear & Radvansky, 2021; Radvansky, Copeland, & Von Hippel, 2010; Radvansky, Copeland, & Zwaan, 2003; Schmalhofer & Glavanov, 1986; Wasiuk, Radvansky, Greene, & Calandruccio, 2021; Zwaan, 1994).

In addition to the memory probes, as a more exploratory component, we assessed viewers’ attitudes for characteristics relevant to each video. For the rideshare video, we used the following scale, “Using a 1 (completely disagree) to 9 (completely agree) scale, please indicate the degree to which you learned the following from the rideshare video.” We asked participants to rate their attitudes on the following two sentences for that video: “It is important to treat others with respect” and “It is important to maintain self-control.” For the council meeting video, using the same response scale, participants rated their attitudes on: “It is important to think carefully before you do anything,” and “Actions have consequences.”

Lastly, for each video, people were asked to rate their confidence in their memories for the videos. They did this using the scale of 1 = low to 9 = high on the following two sentences: “How confident are you in your memory for the rideshare story?” and “How confident are you in your memory for the council meeting story?”

#### Procedure

**Fig. 2:**
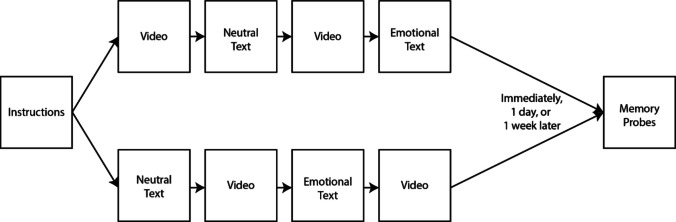
Flow of the study

Participants first consented to be in the study. The flow of study after that is shown in Fig. 2. After being given some instructions, half of the participants first watched a video, then read the text paired with that video, followed by the other video, and then the text paired with that video. For the other half of the participants, the text was presented first and the videos second. The order of which video/text pair was first was counterbalanced across participants. The emotional text version was always second. This was done to avoid priming participants about a possible emotional interpretation of the second video if the emotional version was presented first.

Following the video and the text, people were probed for information regarding their memory of the videos and text. These probes were presented either immediately after (with a slight delay while people read the instructions), 1 day later, or 1 week later. These memory probe questions were followed by the attitude and confidence questions. At the end of the study, participants were told that the text information given was fictitious, and that the researchers had no knowledge of what transpired after the videos.

### Results

The results are divided into two major sections – memory for the video content and memory for the text. Data from all our memory measures were submitted to 3 (Delay: Immediate, 1 day, and 7 days) x 2 (Text Position: before or after) x 2 (Text Valence: Neutral or Emotional) mixed ANOVAs, with the first two variables being between-subjects and the third within-subjects. Recognition memory was assessed in two ways: signal detection analysis and hit rates. Signal detection analyses are common with recognition and provide information about the ability to discriminate old from new information, but they do not provide information the amount of information or the rate of accepting different kinds of information. To this end, we also analyzed the hit rates. We did not analyze false alarm rates because they do not easily fall into our different levels of memory categories. As such they are not informative of processes operating at different levels of representation.

#### Video memory

There are two levels of analysis for video memory: detail and gist. Again, detail memory refers to information that was perceptually available in the video, whereas gist refers to inferences drawn during viewing in order to understand the depicted events.

**Fig. 3:**
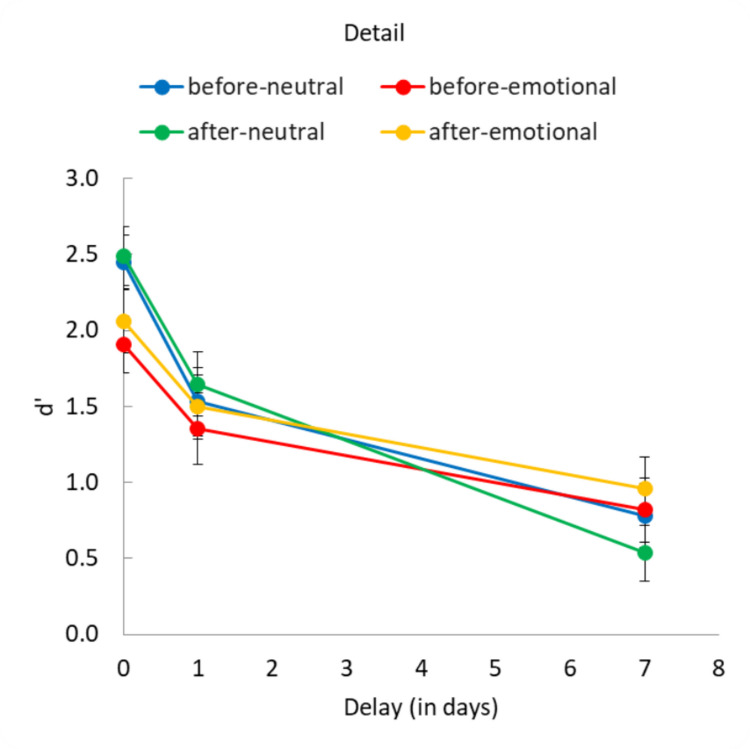
Memory for details of the video using signal detection

The *detail memory signal detection d’ data* are summarized in Fig. 3. There was a main effect of Delay, *F*(2,607) = 51.25,* p* <.001, *η*_*p*_^*2*^ =.14, as expected, with memory becoming worse over time, with significant differences between the immediate and 1-day delays, *F*(2,407) = 23.35,* p* <.001, *η*_*p*_^*2*^ =.05, and the 1-day and 7-day delays, *F*(2,377) = 23.95,* p* <.001, *η*_*p*_^*2*^ =.06. Although the main effect of Text Valence was not significant, *F*(1,607) = 1.44,* p* =.23, *η*_*p*_^*2*^ =.001, there was a significant Delay x Text Valence interaction,* F*(2,607) = 3.36,* p* =.035, *η*_*p*_^*2*^ =.01. Simple effects tests revealed that there was an effect of Text Valence for the immediate memory test, *F(1,230)* = 7.08,* p* =.008, *η*_*p*_^*2*^ =.03, but not for the 1-day and 7-day tests, *F*(1,177) = 0.52,* p* =.47, *η*_*p*_^*2*^ =.003, and *F*(1,200) = 1.36,* p* =.24, *η*_*p*_^*2*^ =.01. Specifically, video detail memory was worse when the accompanying text was emotional, suggesting that this outside information disrupted performance. This is an emotional verbal overshadowing effect. However, this disruption difference was absent over longer periods of time after some forgetting had occurred. Thus, the impact of emotion on memory here was transient, and not long-lasting. None of the other interactions were significant, all *p*s >.10.

**Fig. 4:**
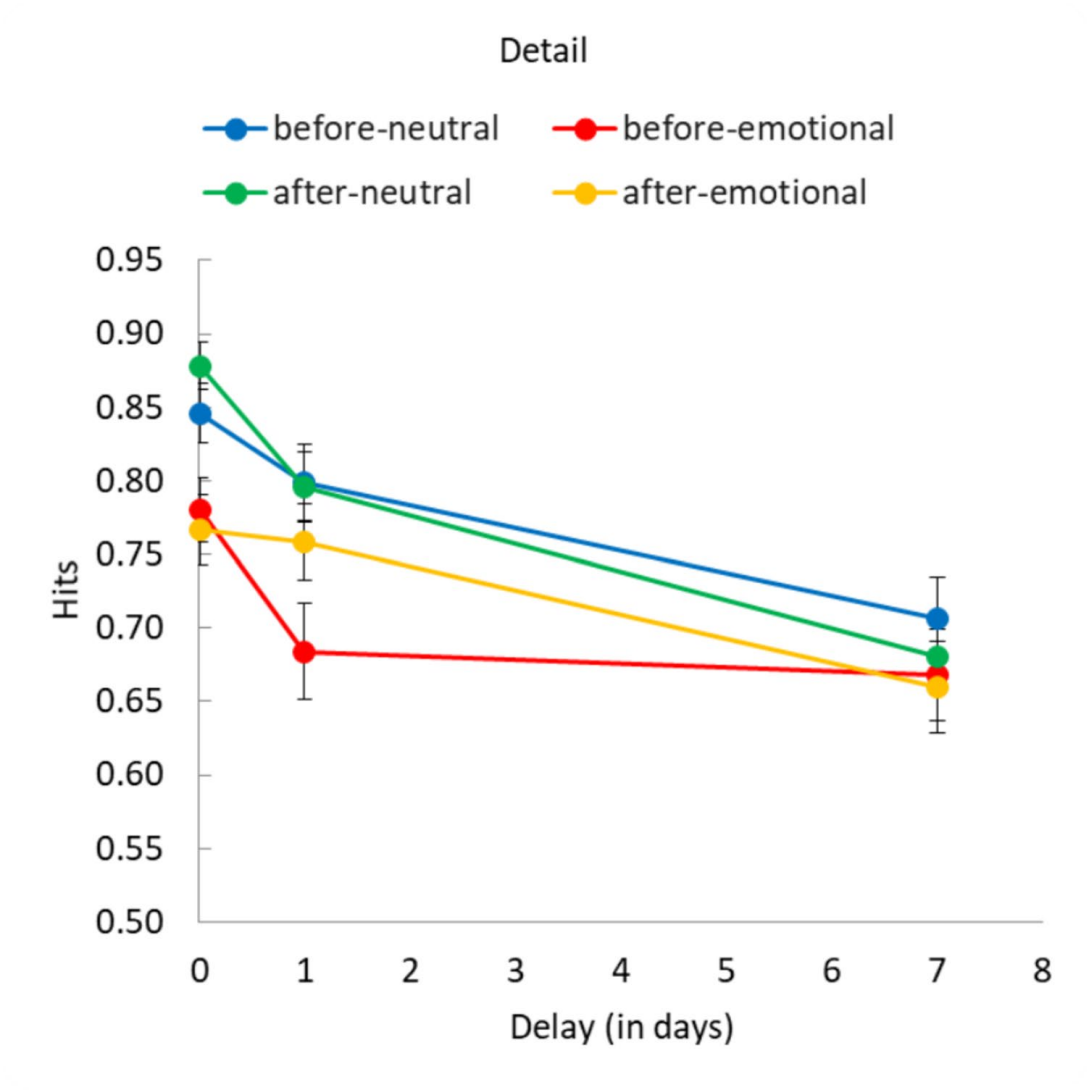
Hit rate memory for details of the video

The *detail memory hit rate data* are summarized in Fig. 4. Not surprisingly, the ANOVA revealed a main effect of Delay, *F*(2,607) = 34.91,* p* <.001, *η*_*p*_^*2*^ =.10, with hit rates declining as more time had elapsed, with significant differences between the immediate and 1-day delays, *F*(2,407) = 12.49,* p* <.001, *η*_*p*_^*2*^ =.03, and the 1-day and 7-day delays, *F*(2,377) = 18.78,* p* <.001, *η*_*p*_^*2*^ =.05. There was also a main effect of Text Valence, *F*(1,607) = 18.46,* p* <.001, *η*_*p*_^*2*^ =.03. Again, there was evidence of an emotional verbal overshadowing effect, with memory for the video being worse when accompanied by emotional (*M* =.50; *SE* =.01) than by neutral text (*M* =.55; *SE* =.01).

**Fig. 5:**
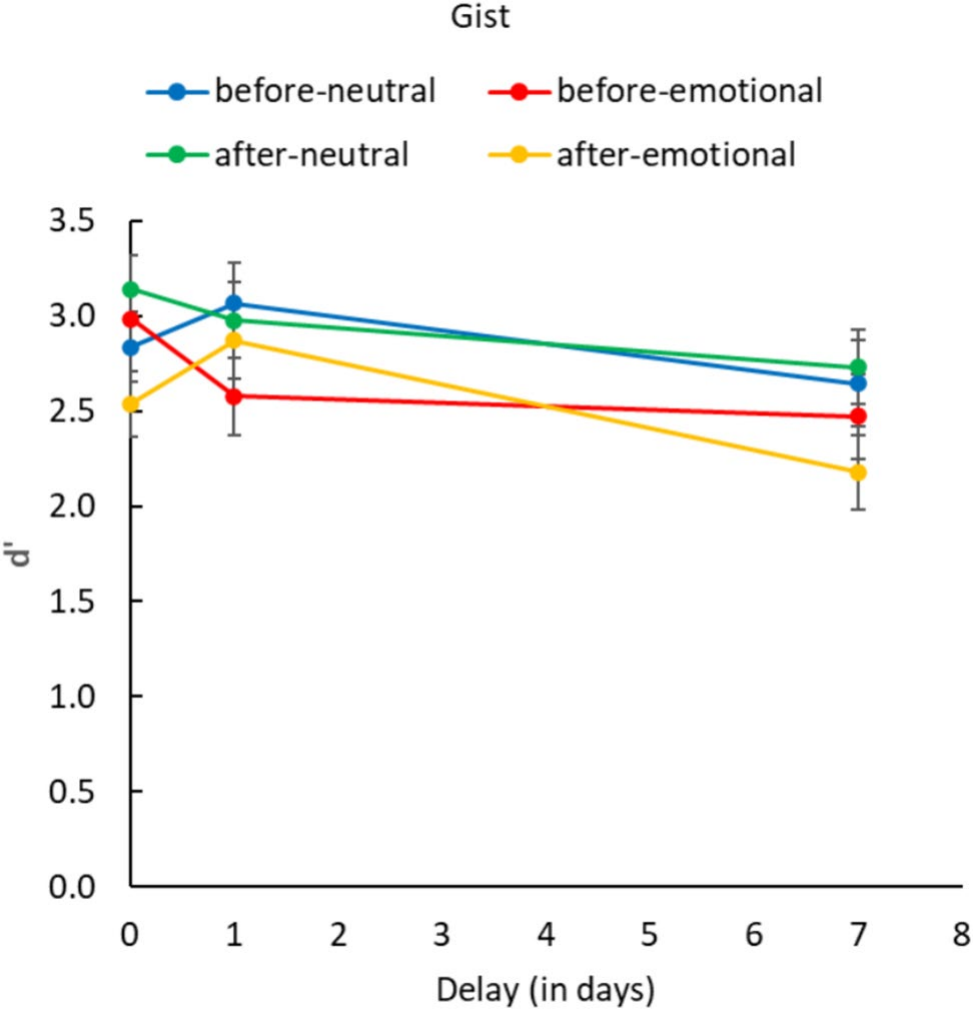
Memory for gist of the video using signal detection

The *gist memory signal detection d’ data* are summarized in Fig. 5. There was a main effect of Delay, *F*(2,607) = 4.32, *p =*.01, *η*_*p*_^*2*^ =.02, as expected, with memory becoming worse over time. That said, there was no difference the immediate and 1-day delays, *F*(2,407) =.00024, *p =*.99, *η*_*p*_^*2*^ <.001, but there was between the 1-day and 7-day delays, *F*(2,377) = 5.68,* p =*.02, *η*_*p*_^*2*^ =.02. Overall, the change in memory over time is strikingly less severe compared to detail memory. This is consistent with other research showing that memory retention at the event model level shows little change (Fisher & Radvansky, 2021).

There was also a main effect of Text Valence, *F*(1,607) = 7.55,* p* =.006, *η*_*p*_^*2*^ =.01, with memory being better when the video was accompanied by Neutral (*M* = 2.90; *SE* =.08) than by Emotional texts (*M* = 2.60; *SE* =.08). Again, this is consistent with an emotional verbal overshadowing effect, this time affecting the gist level of representation.

**Fig. 6:**
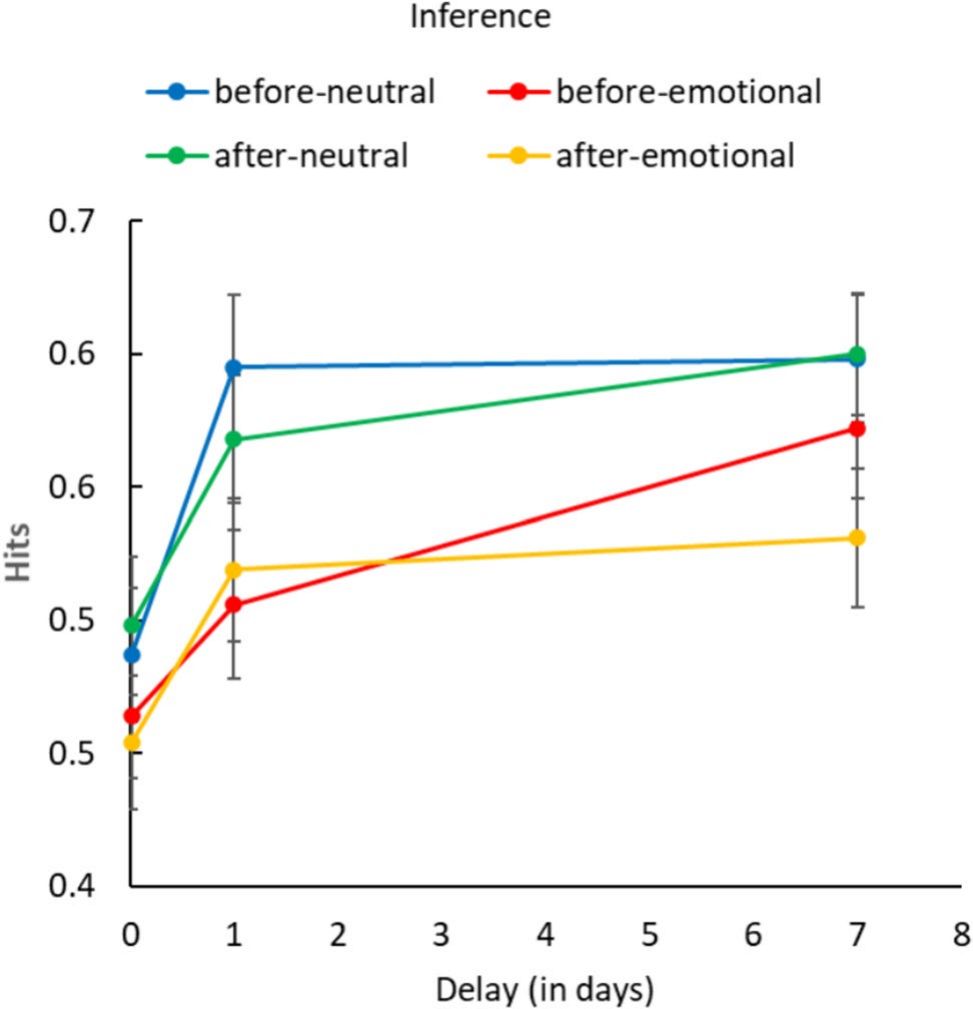
Hit rate memory for inferences consistent with the video

The *gist memory hit data* are summarized in Fig. 6. There was a main effect of Delay, *F*(2,607) = 14.39, *p <*.001, *η*_*p*_^*2*^ =.05, with acceptance of inferences growing larger over time, with a significant difference between the immediate and 1-day delays, *F*(2,407) = 12.28,* p* <.001, *η*_*p*_^*2*^ =.03, but not between the 1-day and 7-day delays, *F*(2,377) = 2.09,* p* =.15, *η*_*p*_^*2*^ =.01.

There was a main effect of Text Valence, *F*(1,607) = 14.94, *p <*.001, *η*_*p*_^*2*^ =.02, with a greater use of inferences when the accompanying text was neutral (*M* =.55; *SE* =.01) rather than emotional (*M* =.50; *SE* =.01). Thus, people were less likely to rely on their memory for the depicted event to respond to memory probes when the accompanying text was emotional. This again is an emotional verbal overshadowing effect.

#### Text memory

**Fig. 7:**
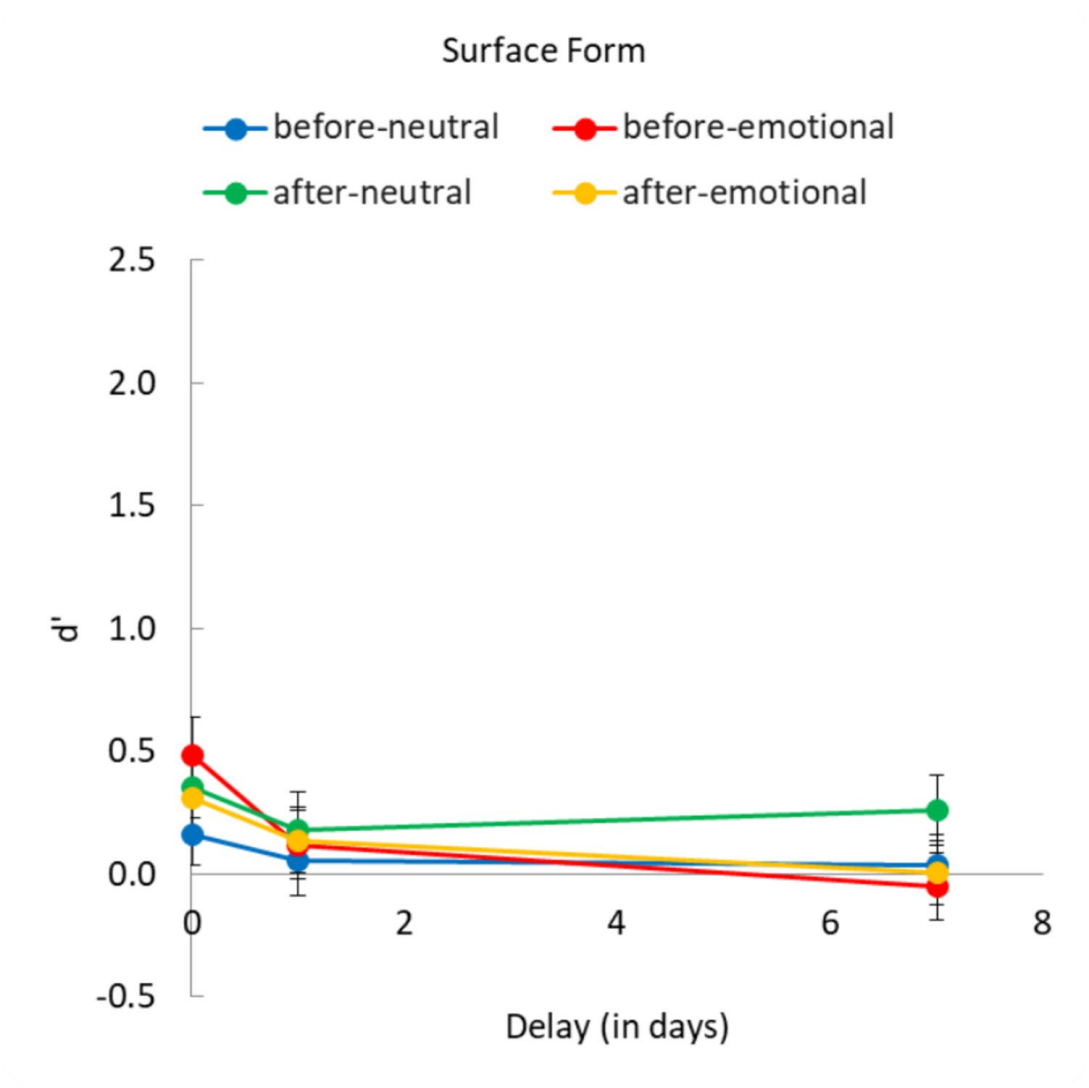
Surface form memory for the text using signal detection

There are three levels of analysis for text memory: surface form, textbase, and event model. These measures are not independent, so they are analyzed separately. The *surface form signal detection d’ memory data* are summarized in Fig. 7. There was a main effect of Delay, *F*(2,607) = 3.97, *p =*.02, *η*_*p*_^*2*^ =.01, as expected, with memory becoming somewhat worse over time, with a marginally significant difference between the immediate and 1-day delays, *F*(2,407) = 3.80, *p =*.05, *η*_*p*_^*2*^ =.01, but not between the 1-day and 7-day delays, *F*(2,377) = 0.33,* p* =.57, *η*_*p*_^*2*^ <.001. Thus, overall, there was very little change over time. To some extent this is expected given that memory for verbatim information is so poor soon after reading (Sachs, 1967). None of the other main effects or interactions were significant, all *p*s >.15.

**Fig. 8:**
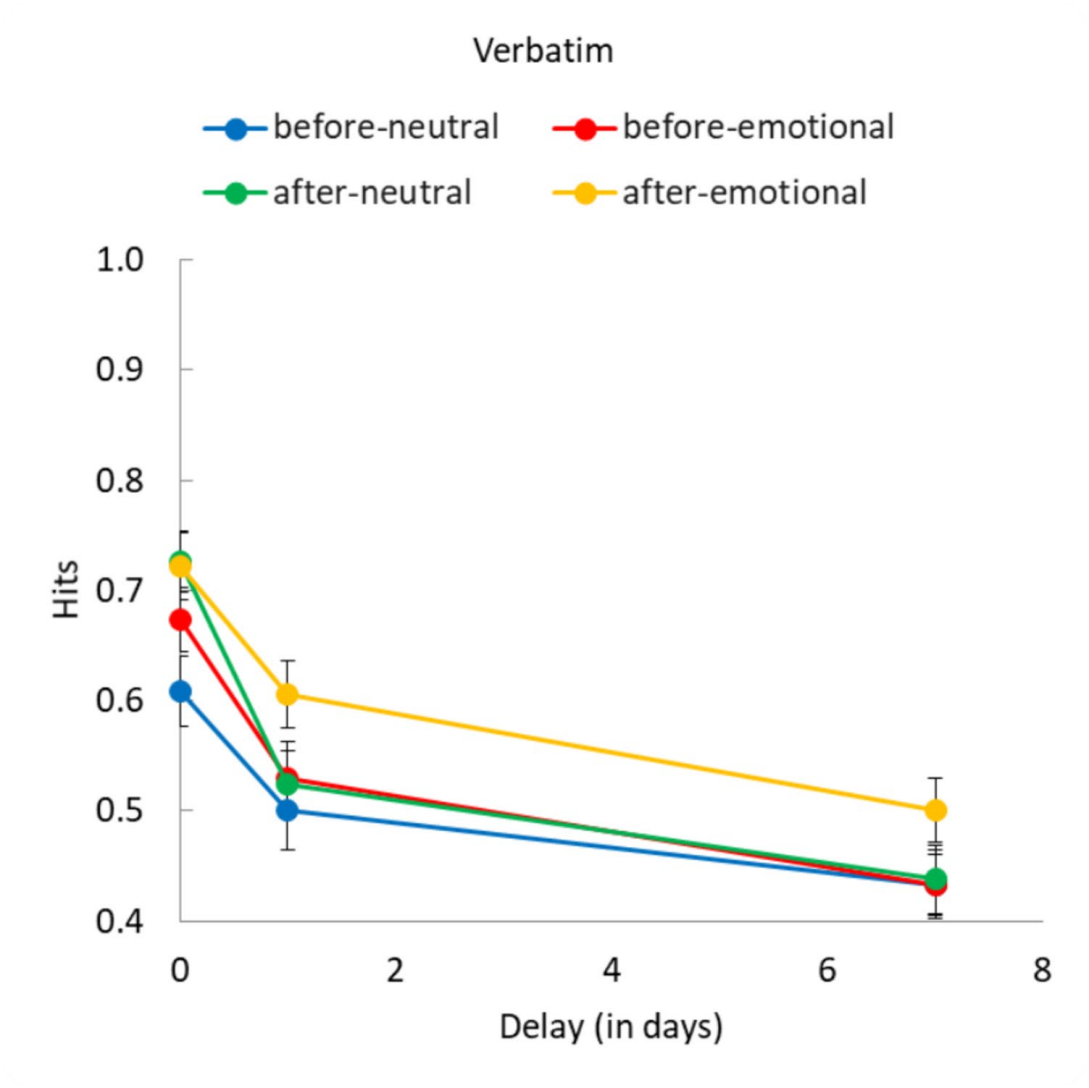
Hit rate memory for the verbatim form of the text

The *verbatim memory hit data* are summarized in Fig. 8. There was also a main effect of Delay, *F*(2,607) = 43.61, *p <*.001, *η*_*p*_^*2*^ =.13, as expected, with worse memory with longer delays, with differences between the immediate and 1-day delays, *F*(2,407) = 28.43,* p* <.001, *η*_*p*_^*2*^ =.07, and the 1-day and 7-day delays, *F*(2,377) = 11.88,* p* <.001, *η*_*p*_^*2*^ =.03. There was also a main effect of Text Valence, *F*(1,607) = 7.98,* p* =.005, *η*_*p*_^*2*^ =.01, with better memory for emotional (*M* =.58; *SE* =.01) than neutral texts (*M* =.54; *SE* =.01). This is a standard finding. There was no interaction with Intensity, *F* < 1.

**Fig. 9:**
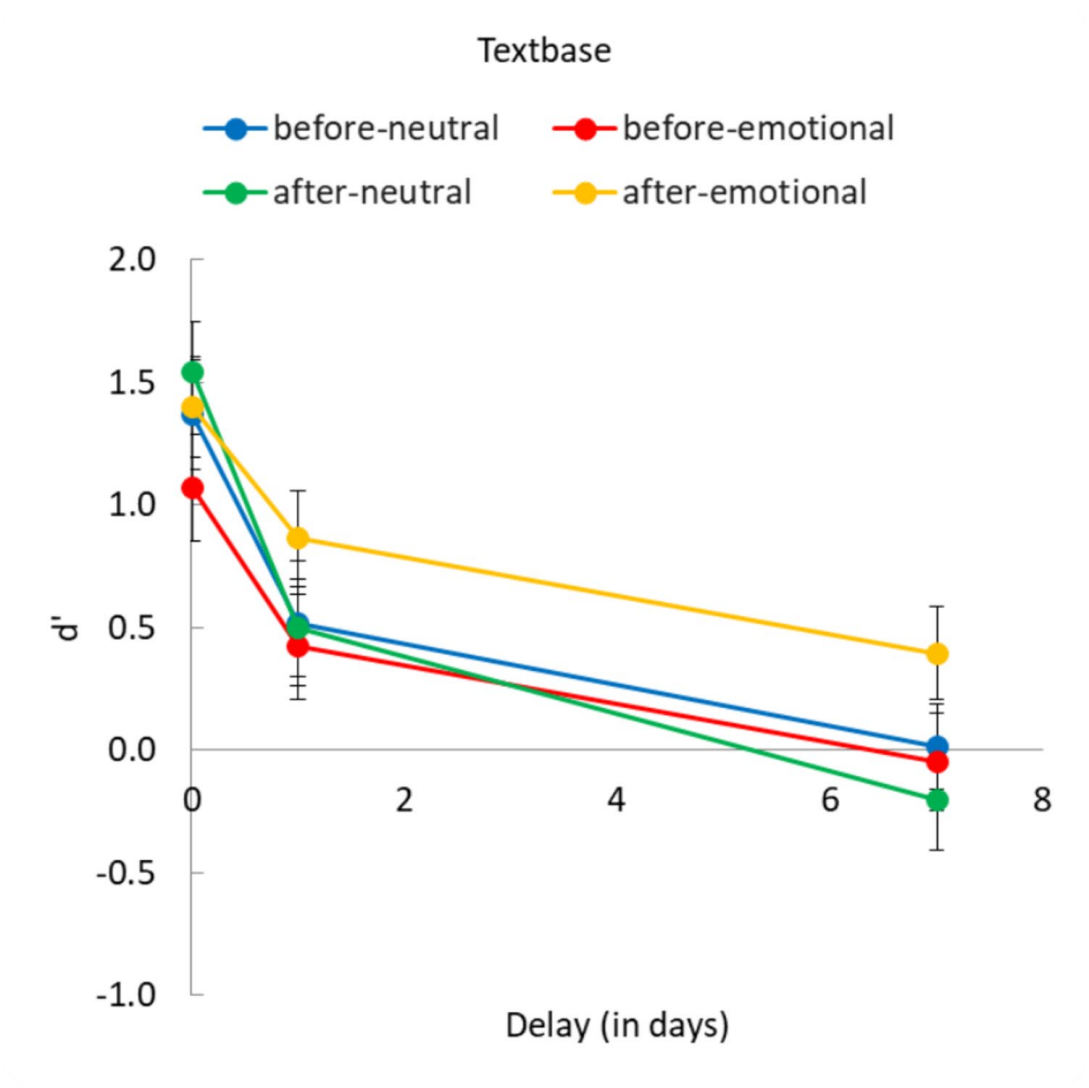
Textbase memory for the text using signal detection

The *textbase signal detection d’ memory data* are summarized in Fig. 9. There was a main effect of Delay, *F*(2,607) = 35.92, *p <*.001, *η*_*p*_^*2*^ =.11, as expected, with memory becoming worse over time, with differences between the immediate and 1-day delays, *F*(2,407) = 21.30,* p* <.001, *η*_*p*_^*2*^ =.05, and the 1-day and 7-day delays, *F*(2,377) = 12.03, *p* <.001, *η*_*p*_^*2*^ =.03. There were no main effects of Text Valence or Text Position, both *F*s < 1. However, there was a marginally significant Text Valence x Text Position interaction, *F*(1,607) = 3.72, *p =*.054, *η*_*p*_^*2*^ =.01. When analyzed separately, when the text came before the video, there was no effect of Text Valence,* F* < 1. However, when the text came after viewing the video, there was a marginally significant main effect of Text Valence, *F*(1, 259) = 3.68, *p =*.06, *η*_*p*_^*2*^ =.01, with better textbase memory when the content was emotional (*M* = 0.84; *SE* =.13) than when it was neutral (*M* = 0.53; *SE* =.13). This is a common finding of better memory when information is emotional rather than neutral.

**Fig. 10:**
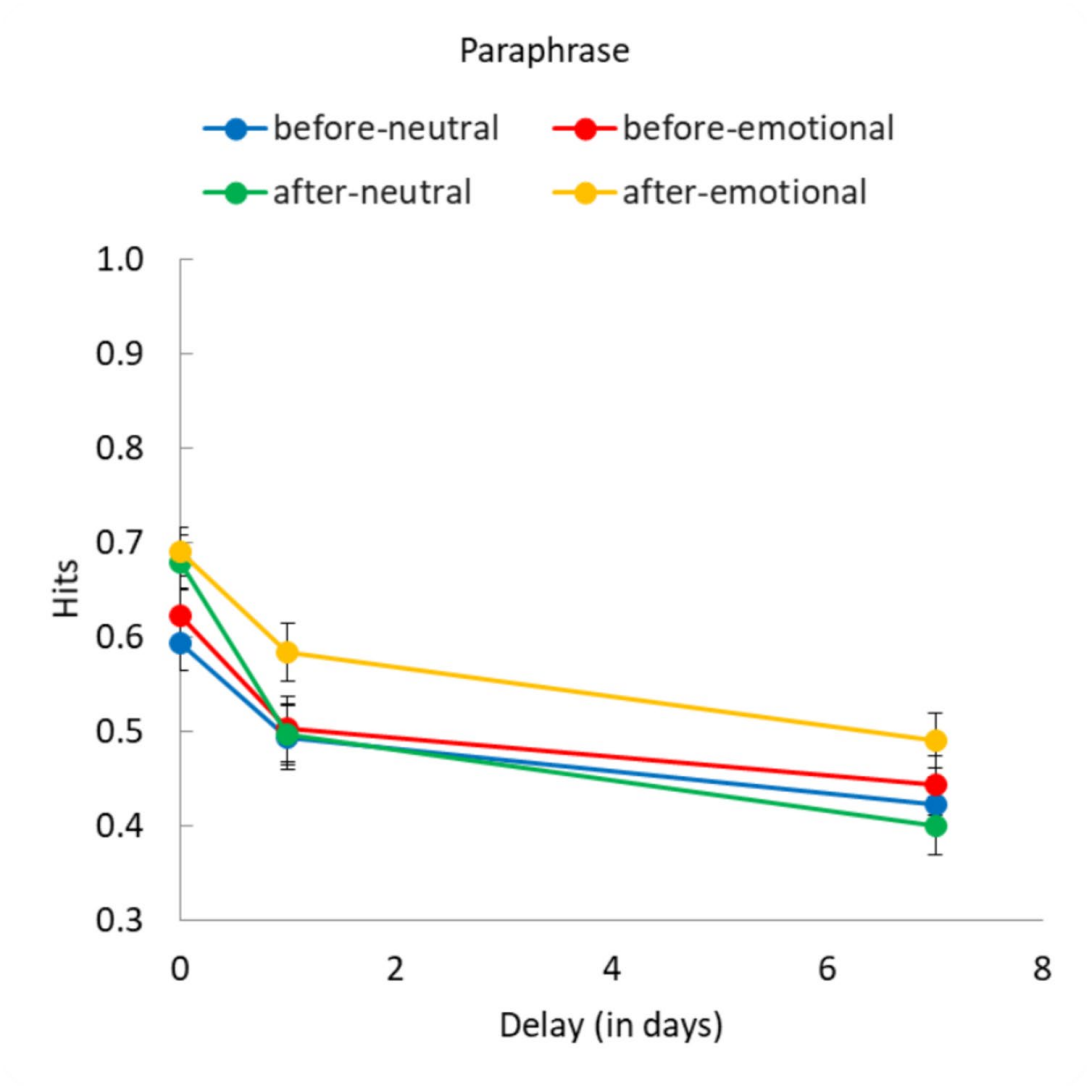
Hit rate memory for paraphrases of the text

The *paraphrase memory hit data* are summarized in Fig. 10. There was a main effect of Delay, *F*(2,607) = 37.53, *p* <.001, *η*_*p*_^*2*^ =.11, as expected, with worse memory with longer delays, with differences between the immediate and 1-day delays, *F*(2,407) = 25.26,* p* <.001, *η*_*p*_^*2*^ =.06, and the 1-day and 7-day delays, *F*(2,377) = 9.73,* p* =.002, *η*_*p*_^*2*^ =.03. There was also a main effect of Text Valence, *F*(1,607) = 8.54, *p =*.004, *η*_*p*_^*2*^ =.01, with a higher rate of “yes” responses for paraphrase probes for emotional than neutral texts. This again is a standard finding of better memory for emotional than for neutral information.

**Fig. 11:**
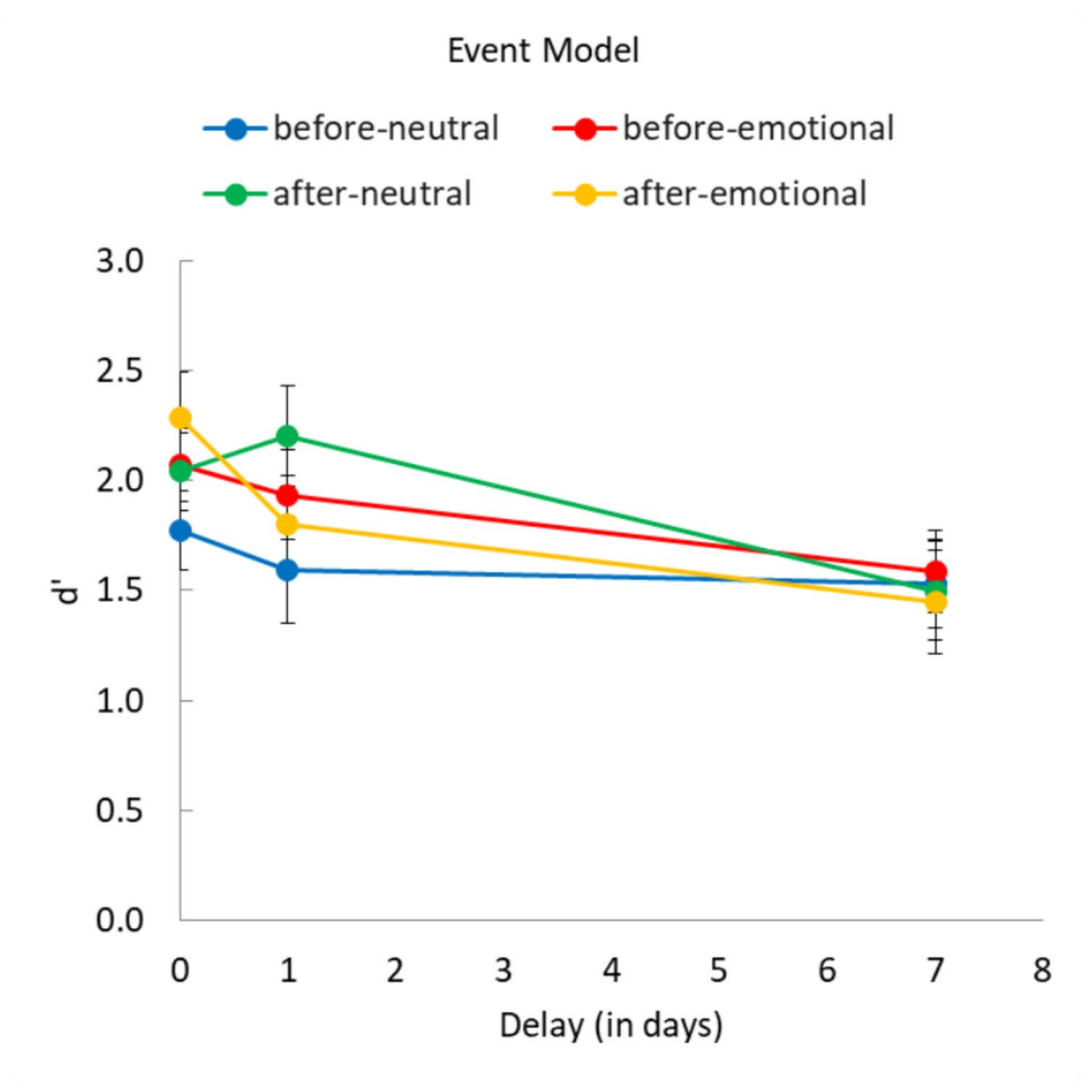
Event model memory for the text using signal detection

The *event model signal detection memory data* are summarized in Fig. 11. There was a main effect of Delay, *F*(2,607) = 6.68,* p* =.001, *η*_*p*_^*2*^ =.02, as expected, with memory becoming worse over time. That said, there was no difference between the immediate and 1-day delays, *F*(2,407) = 1.15,* p* =.28, *η*_*p*_^*2*^ =.003, but there was between the 1-day and 7-day delays, *F*(2,377) = 5.05,* p* =.03, *η*_*p*_^*2*^ =.01. None of the other main effects or interactions were significant (although Text Position, Text Valence, and the three-way interaction are trending in that direction).

**Fig. 12:**
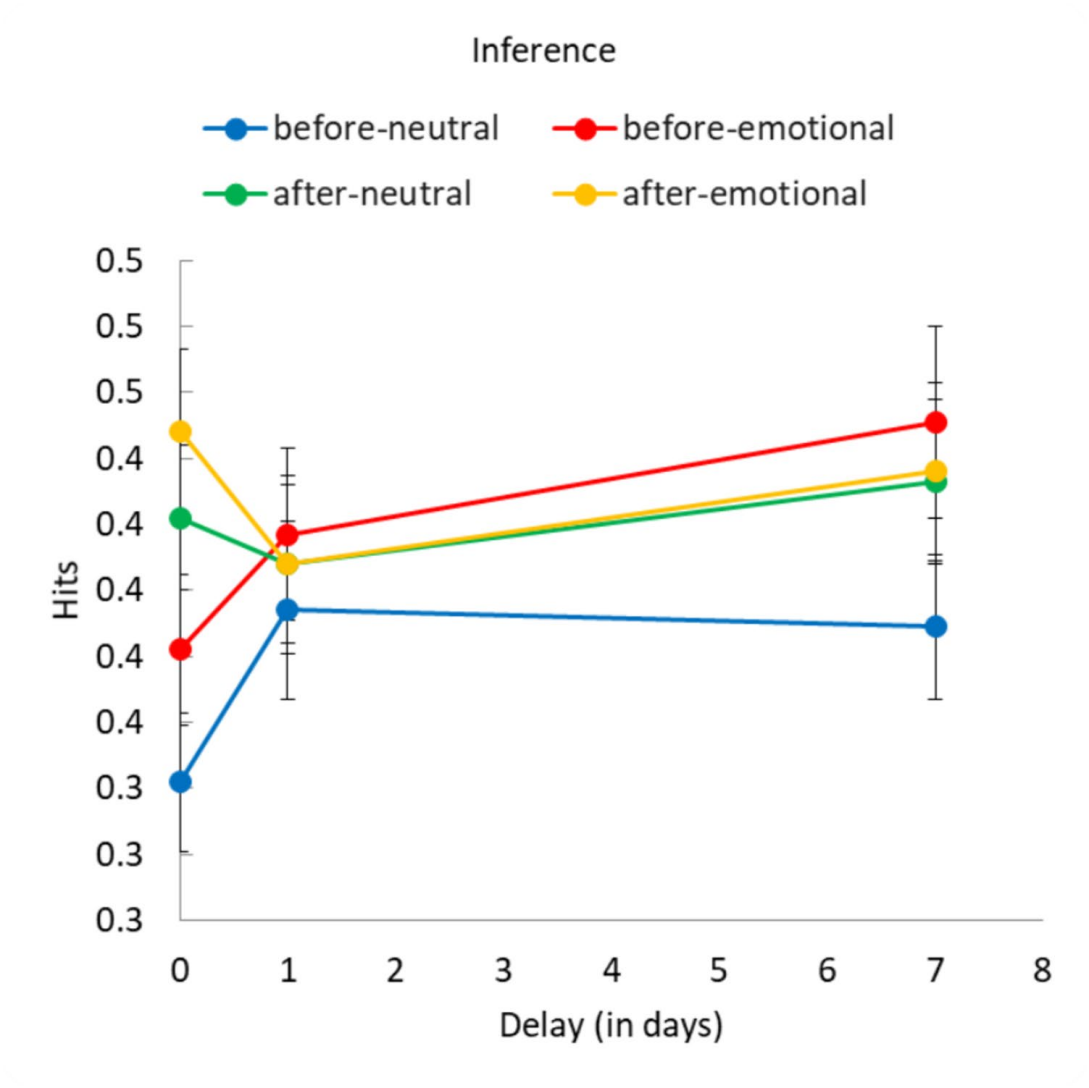
Hit rate memory for inferences of the text

The *inference memory hit data* are summarized in Fig. 12. There was no main effect of Delay, *F*(2,607) = 1.19, *p =*.31, *η*_*p*_^*2*^ =.004, but there was a main effect of Text Valence, *F*(1,607) = 4.34, *p =*.04, *η*_*p*_^*2*^ =.01, with people accepting more inferences for emotional (*M* =.42; *SE* =.01) than neutral texts (*M* =.40; *SE* =.01). There was also a marginally significant effect of Text Position, *F*(2,607) = 3.42,* p* =.065, *η*_*p*_^*2*^ =.04, with people accepting more inferences when the texts came after the video (*M* =.43; *SE* =.01) than when it came before it (*M* =.40; *SE* =.01).

#### Attitude and confidence ratings

In addition to assessing memory for the video and the text, we also assessed attitude and confidence ratings. These are considered here in turn.

As a reminder, for the Lyft video, we asked people to rate, on a 9-point scale, (a) the degree to which “It is important to treat others with respect”; (b) the degree to which “It is important to maintain self-control”; and (c) “How confident are you in your memory for the rideshare story?” Ratings on each of these items were submitted to 3 (Delay) × 2 (Text Position) × 2 (Text Valence: Emotional vs. Neutral) between-subjects ANOVAs.

**Fig. 13:**
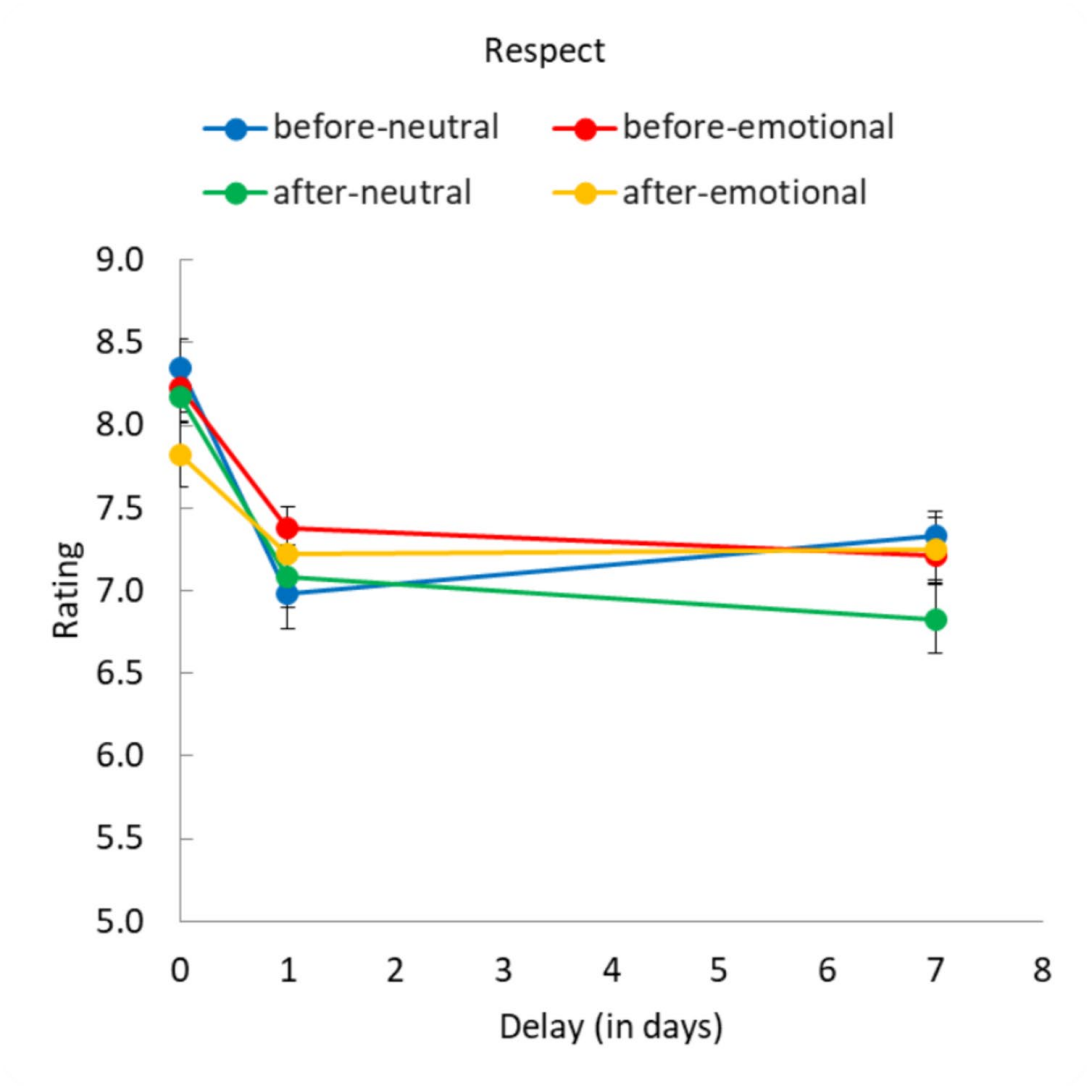
Respect ratings for the Rideshare story

For the respect item, the mean responses are shown in Fig. 13. For these data, there was a main effect of Delay, *F*(2,601) = 43.75, *p <*.001, *η*_*p*_^*2*^ =.13, with ratings declining as more time passed, with the greatest drop being from immediately to a day later. There was a difference between the immediate and 1-day delays, *F*(2,403) = 61.53,* p* <.001, *η*_*p*_^*2*^ =.13, but not the 1-day and 7-day delays, *F*(2,377) = 0.007,* p* =.93, *η*_*p*_^*2*^ <.001. None of the other main effects or interactions were significant, all *p*s >.10. Thus, viewing the video and reading the text increased the likelihood that people would say that they should be more respectful of others, but this influence was temporary. Moreover, the nature of the text that accompanied the video had no effect.

**Fig. 14:**
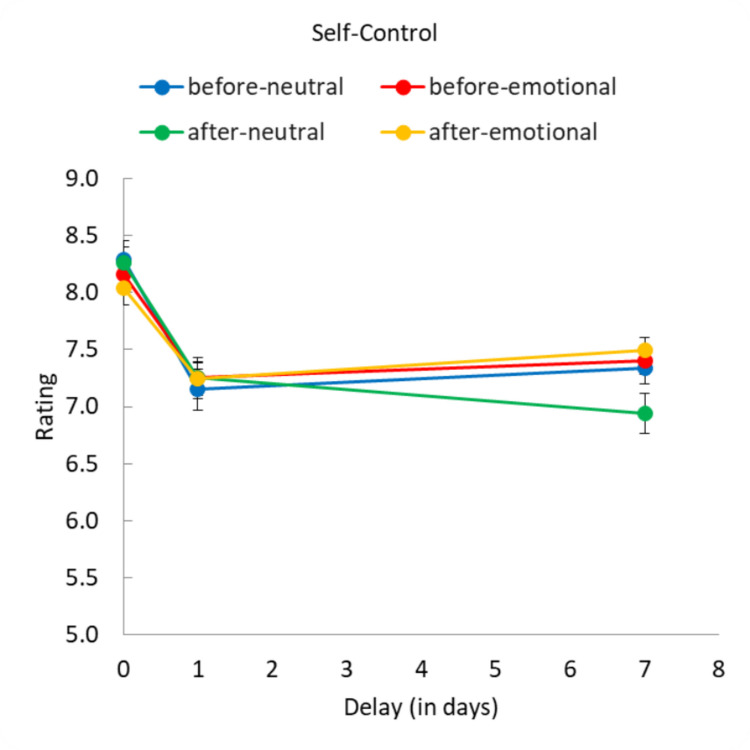
Self-Control ratings for the Rideshare story

For the self-control item, the mean responses are shown in Fig. 14. For these data, there was again a main effect of Delay, *F*(2,601) = 52.52, *p <*.001, *η*_*p*_^*2*^ =.15, with ratings declining as more time passed, with the greatest drop being from immediately to a day later. There was a difference between the immediate and 1-day delays, *F*(2,403) = 73.03,* p* <.001, *η*_*p*_^*2*^ =.15, and the 1-day and 7-day delays, *F*(2,377) = 0.41,* p* =.52, *η*_*p*_^*2*^ =.001. None of the other main effects or interactions were significant, all *p*s >.10. Thus, viewing the video and reading the text increased the likelihood that people would say that people should exhibit self-control, but this influence was temporary. Again, the nature of the text that accompanied the video had no effect.

**Fig. 15:**
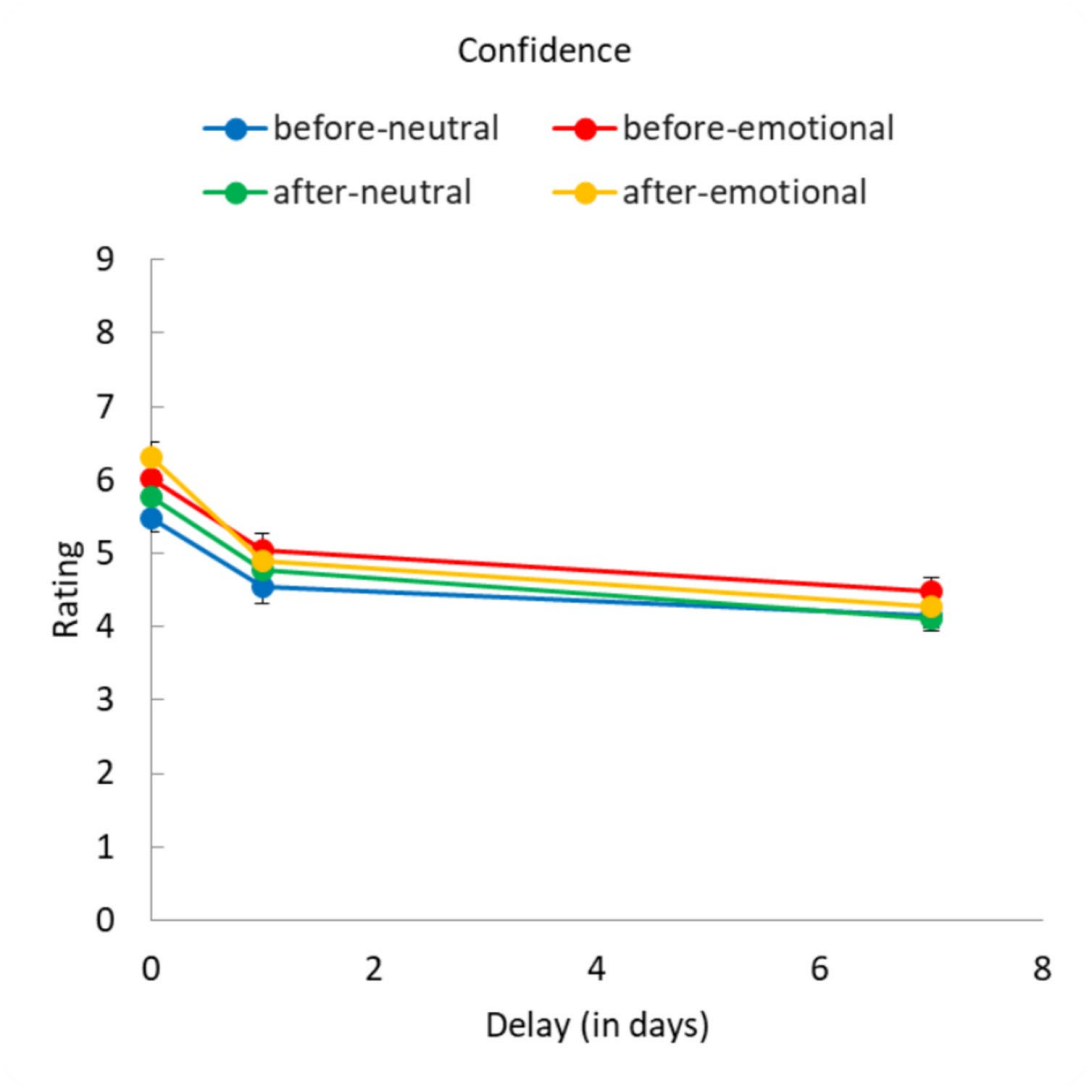
Confidence ratings for the Rideshare story

For the confidence item, the mean responses are shown in Fig. 15. For these data, there was again a main effect of Delay, *F*(2,601) = 69.76, *p <*.001, *η*_*p*_^*2*^ =.19, with ratings declining as more time passed, with the greatest drop being from immediately to a day later. There was a difference between the immediate and 1-day delays, *F*(2,403) = 49.84,* p* <.001, *η*_*p*_^*2*^ =.11, and the 1-day and 7-day delays, *F*(2,377) = 14.97,* p* <.001, *η*_*p*_^*2*^ =.04. There was also a main effect of Text Valence, *F*(1,601) = 9.45, *p =*.002, *η*_*p*_^*2*^ =.02, with people reporting more confidence in their memory when the text was emotional (*M* = 5.17; *SE* =.09) rather than neutral (*M* = 4.81; *SE* =.08). Thus, emotion led people to be confident in their memories. None of the other main effects or interactions were significant, all *p*s >.10. Thus, confidence in memory declined over time, and when the accompanying text was emotional, people consistently had more confidence in their reports.

For the meeting video, we asked people to rate, on a 9-point scale, (a) the degree to which “It is important to think carefully before you do anything”; (b) the degree to which “Actions have consequences”; and (c) “How confident are you in your memory for the council meeting story?” Ratings on each of these items were submitted to 3 (Delay) × 2 (Text Position: Before vs. After) × 2 (Text Valence: Emotional vs. Neutral) between-subjects ANOVAs.

**Fig. 16:**
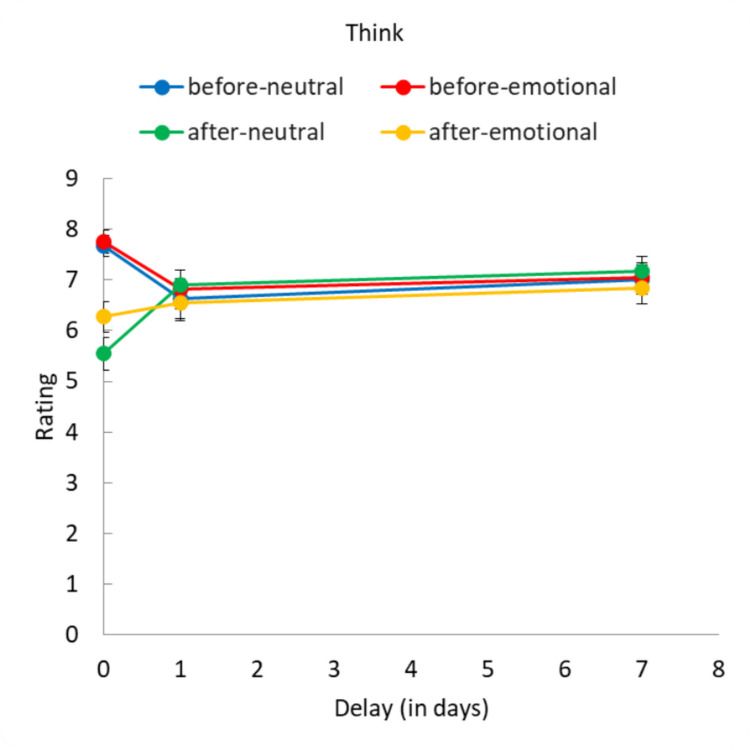
Think carefully ratings for the council meeting story

For the “think carefully” item, the mean responses are shown in Fig. 16. For these data, unlike our other measures, there was no main effect of Delay, *F*(2,601) = 0.93, *p =*.39, *η*_*p*_^*2*^ =.003. However, there was a significant main effect of Text Position, *F*(1,601) = 11.65, *p <.001*, *η*_*p*_^*2*^ =.02, which was modified by a significant Delay × Text Position interaction,* F*(2,601) = 12.50, *p <.001*, *η*_*p*_^*2*^ =.04. As can be seen in Fig. 16, ratings were higher immediately on this item when the text came before, compared to when the text came after,* F*(1,228) = 44.93, *p <.001*, *η*_*p*_^*2*^ =.17. However, this difference was gone by 1 day later, *F*(1,175) = 0.002,* p* =.97, *η*_*p*_^*2*^ <.001. Thus, when the video was more recent, it had a greater impact on “think carefully” ratings, but this influence was transient. None of the other main effects of interactions were significant.

**Fig. 17:**
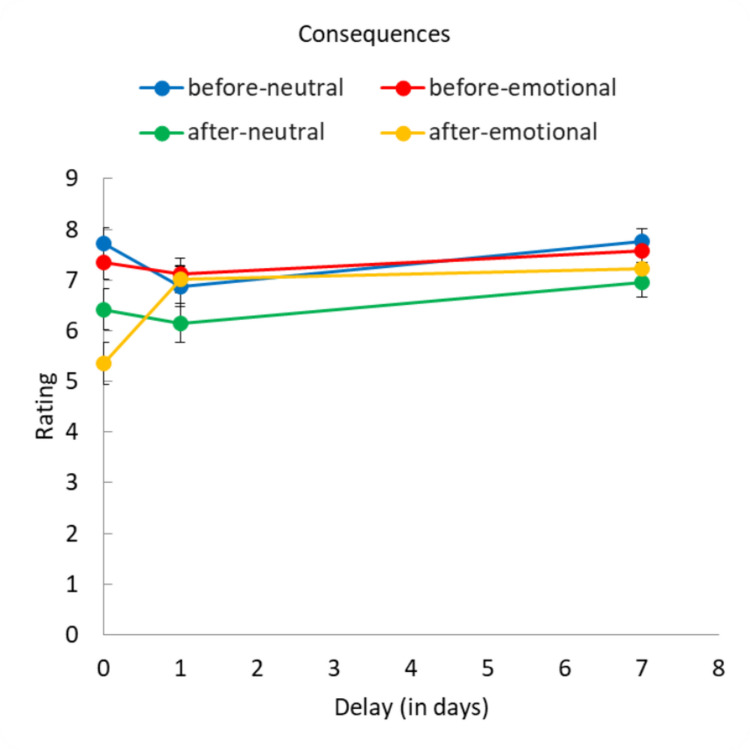
Consequence ratings for the council meeting story

For the “consequences” item, the mean responses are shown in Fig. 17. For these data, there was no main effect of Delay, *F*(2,601) = 4.36, *p =*.01, *η*_*p*_^*2*^ =.02.

**Fig. 18:**
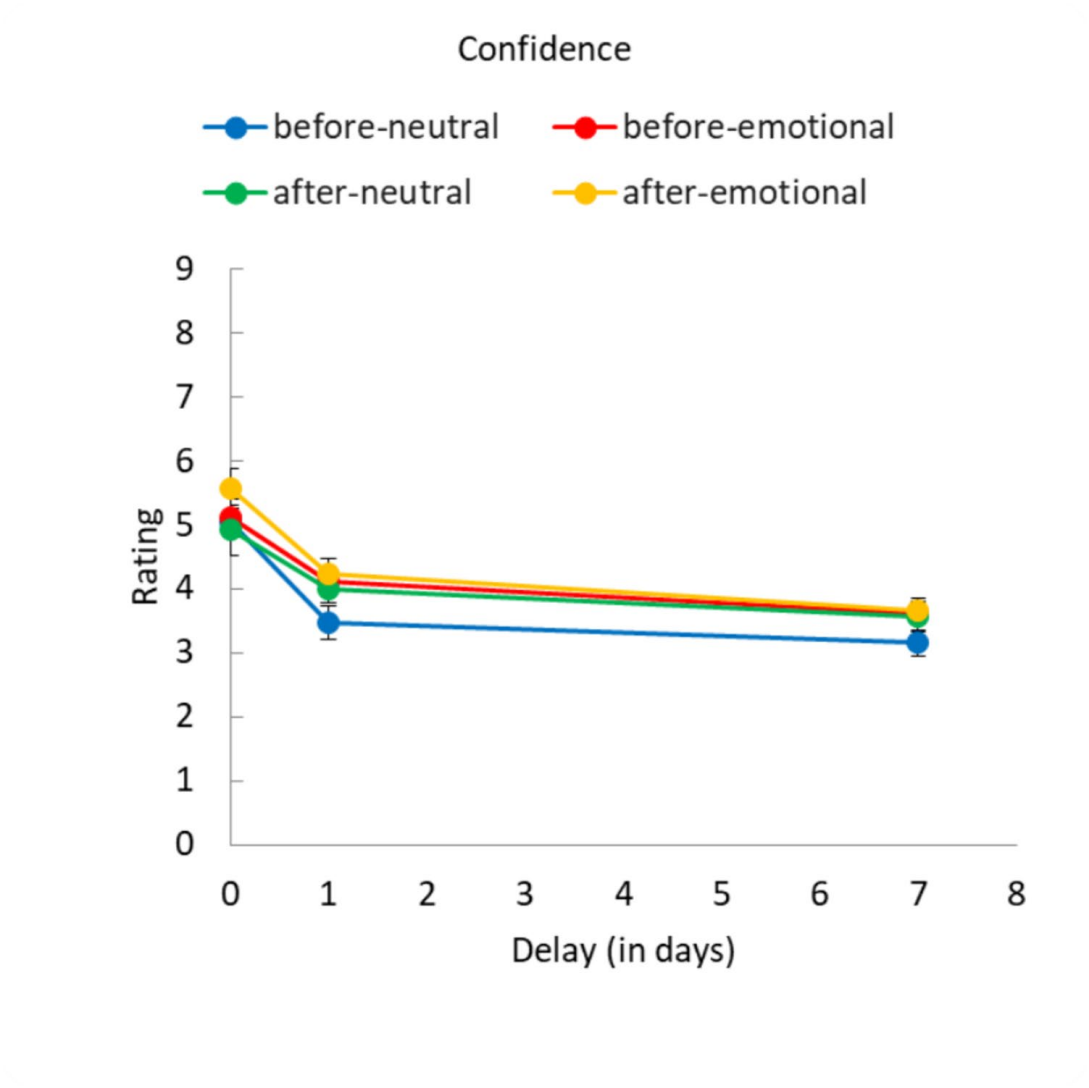
Confidence ratings for the council meeting story

There was also a main effect of Text Position, *F*(1, 601) = 22.48, *p <.001*, *η*_*p*_^*2*^ =.04, which was modified by a significant Delay × Text Position interaction,* F*(2, 601) = 4.66, *p =.01*, *η*_*p*_^*2*^ =.02. As can be seen in Fig. 18, ratings were higher immediately on this item when the text came before, *F*(1,228) = 33.39, *p <.001*, *η*_*p*_^*2*^ =.13, compared to when the text came after,* F*(1,175) = 1.32, *p =.25*, *η*_*p*_^*2*^ =.01. However, this difference was greatly reduced by 1 day later. Thus, when the video was more recent, it had a greater impact on these ratings, but this influence was somewhat transient.

Finally, although the main effect of Text Valence was not significant, *F*(1, 601) = 0.14,* p* =.71, *η*_*p*_^*2*^ =.0002, there was a significant Delay × Text Valence interaction, *F*(1, 601) = 3.65,* p* =.03, *η*_*p*_^*2*^ =.01. Specifically, when ratings were provided immediately, people gave higher ratings when the accompanying text was emotional (*M* = 7.37; *SE* =.20) than when it was neutral (*M* = 6.68; *SE* =.20), *F*(1, 228) = 6.62,* p* =.01, *η*_*p*_^*2*^ =.03. However, there was not a meaningful difference after 1 day between the emotional (*M* = 6.50; *SE* =.22) and the neutral conditions (*M* = 6.91; *SE* =.23), *F*(1, 175) = 1.29,* p* =.26, *η*_*p*_^*2*^ =.01, or after 7 days between the emotional (*M* = 7.33; *SE* =.22) and the neutral conditions (*M* = 7.41; *SE* =.21), *F*(1, 198) = 0.09,* p* =.76, *η*_*p*_^*2*^ <.001. None of the other main effects of interactions were significant.

For the “confidence” item, the mean responses are shown in Fig. 18. For these data, there was a main effect of Delay, *F*(2,601) = 54.50,* p* <.001, *η*_*p*_^*2*^ =.15, with ratings declining as more time passed, with the greatest drop being from immediately to a day later. There was a difference between the immediate and 1-day delays, *F*(2,403) = 42.17, *p* <.001, *η*_*p*_^*2*^ =.10, and the 1-day and 7-day delays, *F*(2,373) = 9.83, *p* =.002, *η*_*p*_^*2*^ =.03. There was also a main effect of Text Position, *F*(1,601) = 5.20, *p =*.02, *η*_*p*_^*2*^ =.01, with people reporting more confidence in their memory when the text came after (*M* = 4.31; *SE* =.09) rather than before (*M* = 4.01; *SE* =.09). Finally, there was also a main effect of Text Valence, *F*(1,601) = 39.11,* p* <.001, *η*_*p*_^*2*^ =.02, with people reporting less confidence in their memory when the text was emotional (*M* = 3.90; *SE* =.09) than when it was neutral (*M* = 4.41; *SE* =.09). Thus, emotion led people to be less confident in their memories, which is opposite to the pattern observed with the rideshare story. None of the interactions were significant, all *p*s >.10.

## General discussion

The current study explored the influence of information encountered outside of an event on later memory for that event. People were asked to view 2-min videos. These were accompanied by fictional texts that elaborated on the information in them. One text had more neutral content, and the other more emotional content. We also manipulated whether the texts were read before or after watching the videos. Finally, recognition memory tests were given for the information in both the witnessed events (the videos) and for the accompanying texts.

Our results revealed that memory for the videos was compromised when the accompanying text had more emotional content. We consider this an emotional verbal overshadowing effect. This implies that an original memory of an experienced event is more likely to be impaired by other content, even when presented in a different modality (i.e., verbal instead of visual), when that other content is emotionally evocative. In some sense, it appears people are interpreting and altering their memories of experienced events through the lens of the emotional external information.

Importantly, the texts that we used contained information about events that occurred subsequent to the video, so that the video itself could be interpreted either neutrally or more negatively. The interpretation could occur either during the video’s presentation if the script was given before the video, or it could have been a post hoc re-interpretation of the video if the script was delivered after the video. We believe that this is a realistic occurrence; for example, how does one remember the details of seeing a person buy goods at a hardware store when the viewer later learns that he was buying materials he later used in a pipe bomb? The answer appears to be that the subsequent, shocking information makes it harder to accurately recall the seemingly innocuous experience of seeing someone buy goods from a hardware store. Although it is unclear how much the “emotional verbal overshadowing effect” results from altered appraisal of the video versus the negative emotion experienced as a result of the negative script, we presume that emotion is driving the impaired memory. The reason for this is that appraisal of the video would be very different depending on when the script was given (i.e., participants who received the negative text *prior to* the video, would appraise the video in a more negative manner; if the text was displayed *after* the video, participants would not watch the video differently at all, but they would *re*-reappraise the video in a more negative context). Still, parsing the potential effects of (re-)appraisal and negative emotion on memory would be a useful next step in this line of research.

This may have implications for memory phenomena such as flashbulb memories. Our memories of some events we experience in life, including public events, do not exist in a vacuum. Instead, they are surrounded by other related memories. Some of these memories provide some interpretive insight into what a given event memory may mean and how to interpret it. This additional interpretative information may come before or after the event. Our memories for surprising and emotional events depends on our attitudes about the subject of the event prior to it occurring, how we interpret those events when we learn of them, and the information that we express and encounter after the fact through discussions with other people and listening and reading news reports (cf. Finkenhauer et al., 1998; Scherer, 2001; Talarico & Rubin, 2007).

Having said this, it should also be noted that this effect, in comparison with traditional verbal overshadowing studies, involved verbal information that was given to the participants rather than verbal information generated by the participants themselves. In comparison to memory for the video, memory for the text itself was better remembered when it contained more emotional content. This is consistent with the common finding in the literature that emotional information is remembered better than neutral information (e.g., Kensinger, 2009; Nadel & Jacobs, 1998; Talmi, 2013). Similarly, for memory for the text itself, the findings are consistent with better gist memory for emotional, negative content (e.g., Adolphs et al., 2005; Bookbinder & Brainerd, 2016; Burke et al., 1992).

Moreover, we did not find any consistent influence of our manipulations on attitudes. Any effects we did observe were transient. Finally, we also observed declines in reported memory confidence over time, but this was also not systematically related to any of our manipulations.

Research on flashbulb memories suggests that encountering emotional information, such as learning about an event, can aid memory for associated, but different, sets of information. For example, when we learn surprising news, we often remember where we were, who we were with, how we learned of the event, and so on. This may not be part of the surprising event, per se, but is associated with its experience. In our study, the emotional information was the accompanying text, and the associated information was the video. In contrast to research on flashbulb memories, we did not see any evidence for a boost for the associated information. Instead, this knowledge was compromised.

Research on altered memories, such as those involved with misleading post-event information, suggests that information learned outside of the original witnessed event can alter the memory for that event. In particular, this information follows the original exposure. However, we are unaware of any research in which the misleading information comes before the witnessed event, given the constraints of how these studies are done. Given this, there seems to be an implicit assumption in this line of work that incorrect information presented after an event would alter memory, but presenting the information before would not. However, we did not see any pattern consistent with such an implicit assumption.

Research on verbal overshadowing suggests that verbal information provided outside of the context of the original event can distort recall or perception of that event. This is because there is some memory competition between the memory of what was seen and the memory for the additional linguistic information. What we observed in the current research is a similar sort of pattern in that, when the additional verbal information was more emotionally intense, there was enhanced memory for that description and poorer memory for the video that was seen. In essence, there seems to be a trade-off, with the memory for the verbal description being heightened at the cost of the memory for what was actually seen. We have labeled this finding an “emotional verbal overshadowing effect” to capture the idea that the emotional content of the language distorts memory for what was seen above and beyond the influence that neutral texts have.

Having made the parallel with research on standard verbal overshadowing, there are some differences which may or may not turn out to be important. First off, the verbal information that typically overshadows the memory for the witnessed event is provided by the participant, not by the researchers, as is the case with our study. Second, the information that is often assessed in competition with the verbal memory is the face of a person (e.g., a perpetrator) in the original event. Finally, in verbal overshadowing studies, the verbal description is often provided after the witnessed event, whereas in our work the description can come before or after the event that was viewed in the video. This highlights the idea that emotional verbal information can impair memory for a witnessed event, even when it is provided from someone else, is not specific to an individual, and can occur both before and after the event that was viewed.

In addition to the memory-related findings, we also assessed how our manipulations influenced various attitudes related to the content of the videos and the texts. We observed attitude ratings consistent with the information highlighted by the scripts. However, these attitude influences were transitory. One day after the stimulus presentation, any influence of the materials on attitudes had vanished. Thus, manipulations of attitudes are short-lived, and do not have lasting effects, even when people are engaged in a task that would have brought to mind the earlier witnessed and read events.

As with any study, there are some limitations to our work. For one, it is not clear to what extent the influence of the additional text on video memory is specific to verbal information, or if similar effects would be observed if the additional information was also presented in a video format. Also, while we were able to compare the influence of the additional text information as a function of whether it came before or after the video, we did not have a condition where there was no additional information. We made this choice because we wanted to keep the overall amount of information the same, which would not be the case if there was not additional information. Thus, if memory were different under those conditions, then no clear conclusions could be drawn as to whether it was due to the absence of additional information, or the lack of general interference from the overall reduced level of information.

## Conclusions

In the present research, we show that the asynchronous presentation of an emotional verbal interpretation of a video degrades recollection of the video itself. Specifically, no matter whether the interpretation was offered before or after, both detail and gist memory for the video were reduced relative to the presentation of a neutral interpretation of the event. We hypothesize that the negative interpretation of the event diverted attention towards the emotional content and away from the actual memory of the video. In the future, researchers may want to determine how the *degree* of the negative interpretation impacts memory for the visual stimulus; for example, does a very negative interpretation degrade memory more than a slightly negative interpretation? Moreover, is memory for video likewise degraded by a *positive* emotional interpretation? The present research has real-world implications: For example, providing a negative interpretation of what an eyewitness observer saw may, in fact, reduce the observer’s ability to accurately recall the event. The influence of the emotional verbal overshadowing effect on real-world scenarios (involving the law, for example) seems important.

## Data Availability

The data and materials are available at https://osf.io/qbw23/

## Footnotes

1 However, it has also been reported that detail information is aided by emotional content (Xu et al., 2011).

2 SAM arousal data were also collected. Self-reported arousal was not reliably influenced by reading the relatively neutral or negative texts.

3 A larger negative score indicated a larger increase in negative affect (NA), whereas a larger positive score indicated a larger increase in positive affect (PA).

## Declarations

### Open practices statement

The data and materials for all experiments are available via the Open Science Framework at https://osf.io/qbw23/ and none of the experiments were preregistered.

## References

[CR1] Adolphs, R., Tranel, D., & Buchanan, T. W. (2005). Amygdala damage impairs emotional memory for gist but not details of complex stimuli. *Nature Neuroscience,**8*(4), 512–518. 15735643 10.1038/nn1413

[CR2] Anderson, A. K., Yamaguchi, Y., Grabski, W., & Lacka, D. (2006). Emotional memories are not all created equal: Evidence for selective memory enhancement. *Learning & Memory,**13*(6), 711–718. 17101871 10.1101/lm.388906 PMC1783624

[CR3] Bohay, M., Blakely, D. P., Tamplin, A. K., & Radvansky, G. A. (2011). Note taking, review, memory and comprehension. *American Journal of Psychology,**124*, 63–73. 21506451 10.5406/amerjpsyc.124.1.0063

[CR4] Bookbinder, S. H., & Brainerd, C. J. (2016). Emotion and false memory: The context–content paradox. *Psychological Bulletin,**142*(12), 1315–1351. 27748610 10.1037/bul0000077

[CR5] Brown, R., & Kulik, J. (1977). Flashbulb memories. *Cognition,**5*, 73–99.

[CR6] Burke, A., Heuer, F., & Reisberg, D. (1992). Remembering emotional events. *Memory & Cognition,**20*, 277–290. 1508053 10.3758/bf03199665

[CR7] Chin, J. M., & Schooler, J. W. (2008). Why do words hurt? Content, process, and criterion shift accounts of verbal overshadowing. *European Journal of Cognitive Psychology,**20*(3), 396–413.

[CR8] Christianson, S. Å., & Loftus, E. F. (1991). Remembering emotional events: The fate of detailed information. *Cognition and Emotion,**5*(2), 81–108.

[CR9] Chronister, S. G., Tamplin, A. K., & Radvansky, G. A. (2022). Assessing levels of narrative memory over time. *American Journal of Psychology,**135*(2), 139–149.

[CR10] Cordonnier, A., & Luminet, O. (2021). Consistency and social identification: A test-retest study of flashbulb memories collected on the day of the 2016 Brussels bombings. *Memory,**29*(3), 305–318. 33620002 10.1080/09658211.2021.1891253

[CR11] Davis, M. (1997). Neurobiology of fear responses: The role of the amygdala. *Journal of Neuropsychiatry,**9*, 382–402. 10.1176/jnp.9.3.382 9276841

[CR12] Ebbinghaus, H., Ruger, H. A., & Bussenius, C. E. (1885/1964). *Memory: A Contribution to Experimental Psychology*. Dover. 10.5214/ans.0972.7531.200408 PMC4117135 25206041

[CR13] Finkenhauer, C., Luminet, O., Gisle, L., El-ahmadi, A., van der Linden, M., & Philipott, P. (1998). Flashbulb memories and the underlying mechanisms of their formation: Toward an emotional-integrative model. *Memory & Cognition,**26*, 516–531. 9610122 10.3758/bf03201160

[CR14] Fisher, J. S., & Radvansky, G. A. (2018). Patterns of forgetting. *Journal of Memory and Language,**102*, 130–141.

[CR15] Fisher, J. S., & Radvansky, G. A. (2021). Degree of learning and linear forgetting. *Quarterly Journal of Experimental Psychology,**75*(8), 1483–1496. 10.1177/17470218211056464 34658270

[CR16] Fletcher, C. R., & Chrysler, S. T. (1990). Surface forms, textbases, and situation models: Recognition memory for three types of textual information. *Discourse Processes,**13*(2), 175–190.

[CR17] Glenberg, A. M., Meyer, M., & Lindem, K. (1987). Mental models contribute to foregrounding during text comprehension. *Journal of Memory and Language,**26*(1), 69–83.

[CR18] Hamann, S. (2001). Cognitive and neural mechanisms of emotional memory. *Trends in Cognitive Sciences,**5*(9), 394–400. 11520704 10.1016/s1364-6613(00)01707-1

[CR19] Hamm, V. P., & Hasher, L. (1992). Age and the availability of inferences. *Psychology and Aging,**7*(1), 56–64. 1558706 10.1037//0882-7974.7.1.56

[CR20] Hirst, W., & Phelps, E. A. (2016). Flashbulb memories. *Current Directions in Psychological Science,**25*(1), 36–41. 26997762 10.1177/0963721415622487 PMC4795959

[CR21] Kensinger, E. A. (2009). *Emotional memory across the adult lifespan*. Psychology Press.

[CR22] Kintsch, W., Welsch, D., Schmalhofer, F., & Zimny, S. (1990). Sentence memory: A theoretical analysis. *Journal of Memory and Language,**29*(2), 133–159.

[CR23] Kleinsmith, L. J., & Kaplan, S. (1963). Paired-associate learning as a function of arousal and interpolated interval. *Journal Of Experimental Psychology,**65*, 190–193. 14033436 10.1037/h0040288

[CR24] Kleinsmith, L. J., & Kaplan, S. (1964). Interaction of arousal and recall interval in nonsense syllable paired-associate learning. *Journal of Experimental Psychology,**67*(2), 124–126. 14114908 10.1037/h0045203

[CR25] Kvavilashvili, L., Mirani, J., Schlagman, S., Foley, K., & Kornbrot, D. E. (2009). Consistency of flashbulb memories of September 11 over long delays: Implications for consolidation and wrong time slice hypotheses. *Journal of Memory and Language,**61*(4), 556–572.

[CR26] LaBar, K. S., & Phelps, E. A. (1998). Arousal-mediated memory consolidation: Role of the medial temporal lobe in humans. *Psychological Science,**9*(6), 490–493.

[CR27] Loftus, E. F. (1979). The malleability of human memory: Information introduced after we view an incident can transform memory. *American Scientist,**67*, 312–320. 475150

[CR28] Loftus, E. F., & Hoffman, H. G. (1989). Misinformation and memory: The creation of new memories. *Journal of Experimental Psychology: General,**118*(1), 100–104. 2522502 10.1037//0096-3445.118.1.100

[CR29] Maio, G. R., Haddock, G., & Verplanken, B. (2019). *The Psychology of Attitudes & Attitude Change* (3rd ed). SAGE.

[CR30] McCloskey, M., Wible, C. G., & Cohen, N. J. (1988). Is there a special flashbulb-memory mechanism? *Journal of Experimental Psychology: General,**117*, 171–181.

[CR31] Meissner, C. A., & Brigham, J. C. (2001). A meta-analysis of the verbal overshadowing effect in face identification. *Applied Cognitive Psychology,**15*(6), 603–616.

[CR32] Mishkin, M., & Appenzeller, T. (1987). The anatomy of memory. *Scientific American,**256*, 80–89. 3589645 10.1038/scientificamerican0687-80

[CR33] Nadel, L., & Jacobs, W. J. (1998). Traumatic memory is special. *Current Directions in Psychological Science,**7*(5), 154–157.

[CR34] Narvaez, D., Radvansky, G. A., Lynchard, N. A., & Copeland, D. E. (2011). Are older adults more attuned to morally charged information? *Experimental Aging Research,**37*(4), 398–434. 21800972 10.1080/0361073X.2011.590756

[CR35] Niedźwieńska, A. (2003). Misleading postevent information and flashbulb memories. *Memory,**11*(6), 549–558. 14982122 10.1080/09658210244000252

[CR36] Odinot, G., & Wolters, G. (2006). Repeated recall, retention interval and the accuracy–confidence relation in eyewitness memory. *Applied Cognitive Psychology,**20*(7), 973–985.

[CR37] O’Rear, A. E., & Radvansky, G. A. (2021). Encoding and referent event influence on retrospective memory. *Quarterly Journal of Experimental Psychology,**74*(6), 1117–1127. 10.1177/1747021820982602 33295234

[CR38] Radvansky, G. A., Copeland, D. E., & Von Hippel, W. (2010). Stereotype activation, inhibition, and aging. *Journal of Experimental Social Psychology,**46*(1), 51–60. 20161549 10.1016/j.jesp.2009.09.010 PMC2805127

[CR39] Radvansky, G. A., Copeland, D. E., & Zwaan, R. A. (2003). Brief report: Aging and functional spatial relations in comprehension and memory. *Psychology and Aging,**18*(1), 161–165. 12641320 10.1037/0882-7974.18.1.161

[CR40] Radvansky, G. A., Doolen, A. C., Pettijohn, K. A., & Ritchey, M. (2022). A new look at memory retention and forgetting. *Journal of Experimental Psychology: Learning, Memory, and Cognition,**48*(11), 1698–1723. 35084927 10.1037/xlm0001110

[CR41] Radvansky, G. A., Parra, D., & Doolen, A. C. (2024). Memory from nonsense syllables to novels: A survey of retention. *Psychonomic Bulletin & Review,**31*(6), 2437–2464. 38714636 10.3758/s13423-024-02514-3 PMC11680664

[CR42] Robinson, M. D., & Johnson, J. T. (1996). Recall memory, recognition memory, and the eyewitness confidence–accuracy correlation. *Journal of Applied Psychology,**81*(5), 587–594.

[CR43] Roediger, H. L., III., & Karpicke, J. D. (2006). Test-enhanced learning: Taking memory tests improves long-term retention. *Psychological Science,**17*(3), 249–255. 16507066 10.1111/j.1467-9280.2006.01693.x

[CR44] Rubin, D. C., & Wenzel, A. E. (1996). One hundred years of forgetting: A quantitative description of retention. *Psychological Review,**103*(4), 734–760.

[CR45] Sachs, J. S. (1967). Recognition memory for syntactic and semantic aspects of connected discourse. *Perception & Psychophysics,**2*(9), 437–442.

[CR46] Scherer, K. R. (2001). Appraisal considered as a process of multilevel sequential checking. In K. R. Scherer, A. Schorr, & T. Johnstone (Eds.), *Appraisal Processes in Emotion* (pp. 92–120). Oxford University Press.

[CR47] Schmalhofer, F., & Glavanov, D. (1986). Three components of understanding a programmer’s manual: Verbatim, propositional, and situational representations. *Journal of Memory and Language,**25*(3), 279–294.

[CR48] Schmolck, H., Buffalo, E. A., & Squire, L. R. (2000). Memory distortions over time: Recollections of the O. J. Simpson trial verdict after 15 and 32 months. *Psychological Science,**11*, 39–45. 11228841 10.1111/1467-9280.00212

[CR49] Schooler, J. W., & Engstler-Schooler, T. Y. (1990). Verbal overshadowing of visual memories: Some things are better left unsaid. *Cognitive Psychology,**22*(1), 36–71. 2295225 10.1016/0010-0285(90)90003-m

[CR50] Staugaard, S. R., & Berntsen, D. (2019). Retrieval intentionality and forgetting: How retention time and cue distinctiveness affect involuntary and voluntary retrieval of episodic memories. *Memory & Cognition,**47*, 893–905. 30725379 10.3758/s13421-019-00904-w

[CR51] Subramanian, R., Shankar, D., Sebe, N., & Melcher, D. (2014). Emotion modulates eye movement patterns and subsequent memory for the gist and details of movie scenes. *Journal of Vision,**14*(3), 31–31. 24672021 10.1167/14.3.31

[CR52] Talarico, J. M., LaBar, K. S., & Rubin, D. C. (2004). Emotional intensity predicts autobiographical memory experience. *Memory & Cognition,**32*(7), 1118–1132. 15813494 10.3758/bf03196886

[CR53] Talarico, J. M., & Rubin, D. C. (2003). Confidence, not consistency, characterizes flashbulb memories. *Psychological Science,**14*, 455–461. 12930476 10.1111/1467-9280.02453

[CR54] Talarico, J. M., & Rubin, D. C. (2007). Flashbulb memories are special after all; in phenomenology, not accuracy. *Applied Cognitive Psychology,**21*(5), 557–578.

[CR55] Talmi, D. (2013). Enhanced emotional memory: Cognitive and neural mechanisms. *Current Directions in Psychological Science,**22*, 430–436.

[CR56] Wasiuk, P. A., Radvansky, G. A., Greene, R. L., & Calandruccio, L. (2021). Spoken narrative comprehension for young adult listeners: Effects of competing voices and noise. *International Journal of Audiology,**60*(9), 711–722. 33586551 10.1080/14992027.2021.1878397

[CR57] Wixted, J. T., & Ebbesen, E. B. (1991). On the form of forgetting. *Psychological Science,**2*(6), 409–415.

[CR58] Wixted, J. T., & Wells, G. L. (2017). The relationship between eyewitness confidence and identification accuracy: A new synthesis. *Psychological Science in the Public Interest,**18*(1), 10–65. 28395650 10.1177/1529100616686966

[CR59] Xu, X., Zhao, Y., Zhao, P., & Yang, J. (2011). Effects of level of processing on emotional memory: Gist and details. *Cognition and Emotion,**25*(1), 53–72. 21432656 10.1080/02699931003633805

[CR60] Zacks, R. T., Hasher, L., Doren, B., Hamm, V., & Attig, M. S. (1987). Encoding and memory of explicit and implicit information. *Journal of Gerontology,**42*(4), 418–422. 3598090 10.1093/geronj/42.4.418

[CR61] Zaragoza, M. S., & Koshmider, J. W. (1989). Misled subjects may know more than their performance implies. *Journal of Experimental Psychology: Learning, Memory, and Cognition,**15*, 246–255. 2522514 10.1037//0278-7393.15.2.246

[CR62] Zwaan, R. A. (1994). Effect of genre expectations on text comprehension. *Journal of Experimental Psychology: Learning, Memory, and Cognition,**20*(4), 920–933.

